# Bim Inhibits Autophagy by Recruiting Beclin 1 to Microtubules

**DOI:** 10.1016/j.molcel.2012.05.040

**Published:** 2012-08-10

**Authors:** Shouqing Luo, Moises Garcia-Arencibia, Rui Zhao, Claudia Puri, Pearl P.C. Toh, Oana Sadiq, David C. Rubinsztein

**Affiliations:** 1 Department of Medical Genetics, Cambridge Institute for Medical Research, University of Cambridge, Hills Road, Cambridge CB2 0XY, UK; 2 Laboratory of Lymphocyte Signalling and Development, Molecular Immunology Programme, The Babraham Institute, Babraham, Cambridge CB22 3AT, UK

## Abstract

Bim is a proapoptotic BH3-only Bcl-2 family member. In response to death stimuli, Bim dissociates from the dynein light chain 1 (DYNLL1/LC8), where it is inactive, and can then initiate Bax/Bak-mediated mitochondria-dependent apoptosis. We found that Bim depletion increases autophagosome synthesis in cells and in vivo, and this effect is inhibited by overexpression of cell death-deficient Bim. Bim inhibits autophagy by interacting with Beclin 1, an autophagy regulator, and this interaction is facilitated by LC8. Bim bridges the Beclin 1-LC8 interaction and thereby inhibits autophagy by mislocalizing Beclin 1 to the dynein motor complex. Starvation, an autophagic stimulus, induces Bim phosphorylation, which abrogates LC8 binding to Bim, leading to dissociation of Bim and Beclin 1. Our data suggest that Bim switches locations between apoptosis-inactive/autophagy-inhibitory and apoptosis-active/autophagy-permissive sites.

## Graphical abstract

**Graphical Abstract:**

**Figure:**
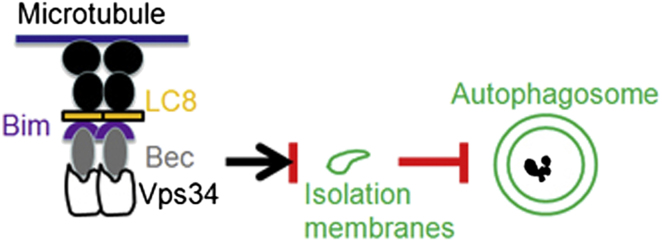

**Highlights:**

► Bim negatively regulates autophagy in cell culture and in vivo ► Bim inhibits autophagosome formation by interacting with Beclin 1 ► Bim inhibits autophagy by mislocalizing Beclin 1 from the ER to microtubules ► Starvation induces autophagy and dissociates the Bim-Beclin1 interaction

## Introduction

The Bcl-2 family includes three main subclasses: antiapoptotic proteins including Bcl-2, Bcl-xL, Bcl-w, MCL-1, and A1 that possess multiple Bcl-2 homology (BH) domains; multidomain proapoptotic proteins that include Bax and Bak, which have BH1-3 domains; and BH3-only proapoptotic proteins, including Bim, Bid, Bad, Noxa, Puma, Bmf, Bik, Bnip3, and Hrk (Adams and Cory, 1998). In viable cells, Bax and Bak are monomeric and inactive. In response to death stimuli, these proteins quickly homooligomerize (Wei et al., 2000). In the absence of both Bax and Bak, cells are resistant to a wide variety of apoptotic stimuli, including UV irradiation, TNF-α, endoplasmic reticulum (ER) stress, and BH3-only proteins (Wei et al., 2001).

Bim has three different splicing isoforms, namely BimEL (the major isoform), BimL, and BimS (O'Connor et al., 1998). In healthy cells, Bim is inactivated by its interaction with dynein light chain 1 (DYNLL1/LC8), which recruits it to the microtubule-based dynein motor complex (Puthalakath et al., 1999). After cells are exposed to apoptotic stimuli, Bim is phosphorylated by JNK at T116, which causes Bim dissociation from LC8 and the dynein motor complex (Lei and Davis, 2003). Bim is essential for apoptosis induced by certain stimuli in cell culture, like cytokine deprivation and ER stress (Puthalakath et al., 2007), and for hematopoietic cell homeostasis in vivo (Bouillet et al., 2002). Interestingly, Bim does not have an evolutionary relationship with the ancient Bcl-2 family, and it may have acquired its BH3 motif by convergent evolution (Aouacheria et al., 2005). This suggests that Bim might have other functions beyond apoptosis.

Macroautophagy (hereafter called autophagy), is a bulk degradation system that mediates clearance of cytoplasmic proteins, certain pathogens and organelles like mitochondria. Double-membraned autophagosomes form randomly in the cytoplasm where they engulf cytoplasmic contents. They are then trafficked to lysosomes where their contents are degraded after fusion (Klionsky et al., 2008). The target of rapamycin (TOR) kinase negatively regulates autophagosome formation under nutrient replete conditions (Lum et al., 2005). Autophagy is activated by starvation via multiple signals, including mTOR inhibition, and AMP-activated protein kinase activation (Inoki et al., 2003).

Beclin 1, a Bcl-2-binding protein (Liang et al., 1998), is an essential autophagy protein that is found in different complexes. Complex I, containing Vps34 (a class III PI-3 kinase that generates PtdIns3P), Vps15, Beclin 1, and Atg14, is required for autophagosome formation (Itakura et al., 2008), while another complex containing UVRAG and Rubicon regulates autophagosome maturation (Matsunaga et al., 2009; Zhong et al., 2009). Structural studies suggest that Beclin 1 is a BH3-only member of the Bcl-2 family and that its BH3 motif mediates its interaction with Bcl-2/Bcl-xL (Oberstein et al., 2007). Bcl-2 and Bcl-xL inhibit autophagy via their interactions with Beclin 1 (Maiuri et al., 2007; Pattingre et al., 2005). The ability of Bcl-2 to inhibit autophagy via Beclin 1 binding is abrogated by JNK1 phosphorylation, which is activated and induces autophagy during starvation (Wei et al., 2008). Bad disrupts the Beclin 1-Bcl-xL interaction and enhances autophagy (Maiuri et al., 2007).

## Results

### Bim Directly Interacts with Beclin 1

**Figure 1:**
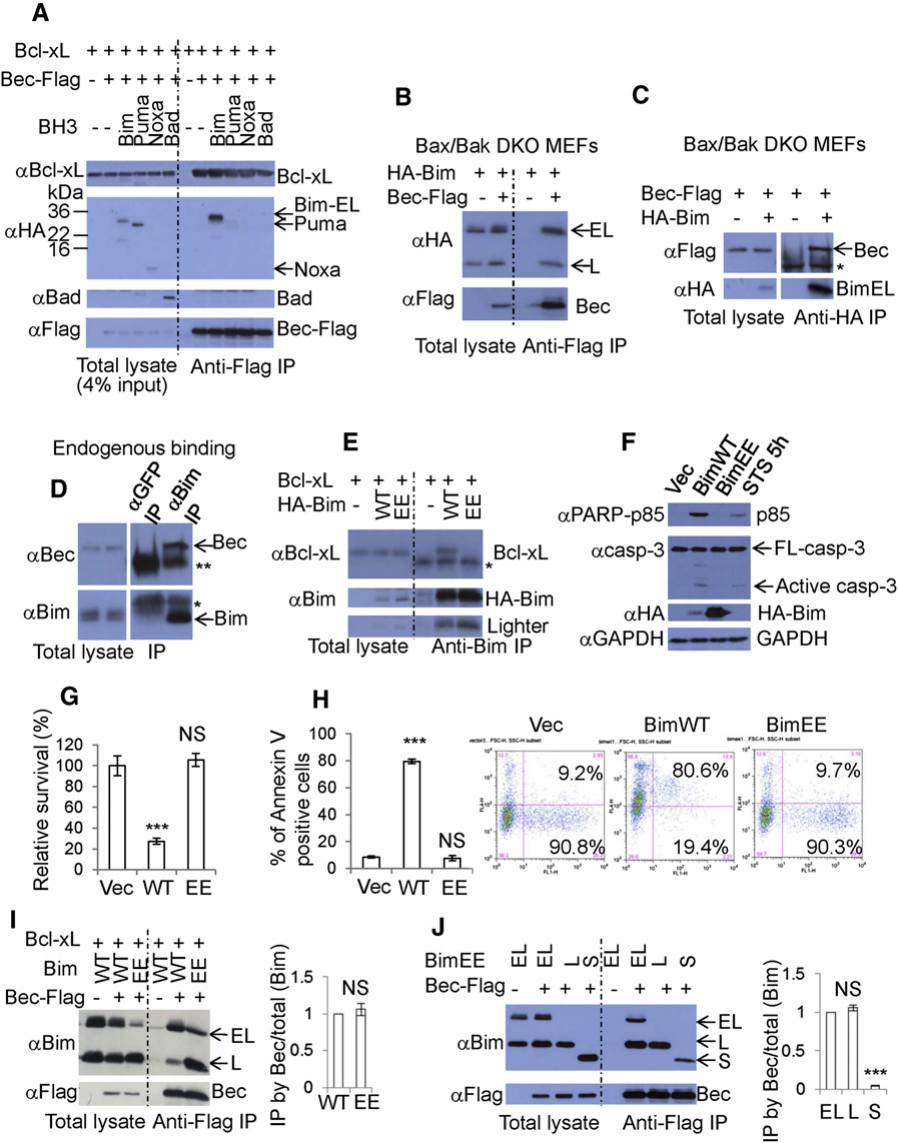
Bim Physically Interacts with Beclin 1 Independently of the Bim BH3 Domain (A) Bcl-xL was cotransfected into HeLa cells with empty vector (IP negative control), Beclin 1-Flag, Beclin 1-Flag/HA-Bim(EL), Beclin 1-Flag/HA-Puma, Beclin 1-Flag/HA-Noxa, or Beclin 1-Flag/Bad. Anti-Flag (M2) was used for immunoprecipitation, and proteins were detected with anti-Bcl-xL, anti-HA, or anti-Bad, anti-Flag (Rabbit). (B) HA-Bim (wild-type) was cotransfected into *Bax/Bak* double-knockout (DKO) mouse embryonic fibroblasts (MEFs) with empty vector (IP negative control) or Beclin 1-Flag. Immunoprecipitations were performed as in (A). (C) Beclin 1-Flag was cotransfected into *Bax/Bak* DKO MEFs with empty vector (IP negative control) or HA-Bim (wild-type). Anti-HA was used for immunoprecipitation. ^∗^, antibody heavy chain. (D) HeLa cell lysates were subjected to GFP antibody (negative control) and anti-Bim antibody immunoprecipitation. Proteins were detected with anti-Beclin 1 and anti-Bim. ^∗∗^, antibody heavy chain; ^∗^, antibody light chain. (E) Bcl-xL was cotransfected into HeLa cells with empty vector (IP negative control), wild-type HA-Bim(EL), or HA-Bim L152E F159E [Bim(EL)EE]. Anti-Bim was used for immunoprecipitation. ^∗^, antibody light chain. (F) HeLa cells were transfected with vector, wild-type HA-Bim, or HA-BimEE. Lysates were probed with the indicated antibodies. HeLa cells treated with staurosporine (STS) for 5 hr were a positive control. Note that wild-type Bim causes apoptosis, which reduces its levels compared to BimEE. (G) Vector, wild-type Bim(EL), or Bim(EL) L152E F159E (BimEE) was transfected into HeLa cells. Transfection efficiency was >90%. After 20 hr, cell viability was determined by measurement pf ATP levels. Data are shown as mean ± SD. ^∗∗∗^p < 0.001. (H) HeLa cells were transfected with vector/GFP (3:1), wild-type HA-Bim/GFP (3:1), or HA-BimEE/GFP (3:1) (to ensure that Bim-transfected cells also contain GFP). After 16 hr, cells were stained with Annexin V-APC and analyzed by FACS. The percentage of APC and GFP double-positive cells/GFP-positive cells are shown. Data are shown as mean ± SD. ^∗∗∗^p < 0.001. (I) Bim(EL)/Bcl-xL were cotransfected into HeLa cells with empty vector (IP negative control) or Beclin 1-Flag; Bim(EL)-EE/Bcl-xL were also cotransfected into HeLa cells with Beclin 1-Flag. Anti-Flag (M2) was used for immunoprecipitation. Data are shown as mean ± SD. NS, not significant. (J) BimEL-EE (EL)/vector (IP negative control), BimEL-EE (EL)/Beclin 1-Flag, BimL-EE (L)/Beclin 1-Flag, and BimS-EE (S)/Beclin 1-Flag were transfected into HeLa cells. Anti-Flag was used for immunoprecipitation. Data are shown as mean ± SD. ^∗∗∗^p < 0.001; NS, not significant. See also Figure S1.

We initially tested whether different BH3-only proteins impaired Beclin 1-Bcl-2/xL interactions. Consistent with previous data (Maiuri et al., 2007), Bad reduced the amounts of Bcl-xL pulled down by Beclin 1, while Puma and Noxa had modest effects (Figure 1A). However, Bim did not affect the Bcl-xL-Beclin 1 interaction (Figure 1A). Beclin 1 did not compromise interactions between Bcl-xL and a series of BH3-only proteins or Bak (Figure S1A available online). Unexpectedly, we observed that Bim was pulled down by Beclin 1, or vice versa, while other BH3-only proteins, Puma, Noxa, and Bad, were not pulled down by Beclin 1, or vice versa (Figures 1A and S1A). Like others, we observed that BimEL overexpression frequently gives rise to both BimEL and a BimL-sized protein. This has been proposed to be BimL due to alternative splicing (U et al., 2001).

We then tested whether the Bim-Beclin 1 interaction was dependent on Bcl-xL, since both Bim and Beclin 1 physically interact with Bcl-xL. We generated a series of Beclin 1 variants and found that all bound Bim, but this was not the case for Bcl-xL, suggesting that the Bim-Beclin 1 interaction does not require Bcl-xL-Beclin 1 binding (Figures S1B and S1C). Furthermore, Bec126 (aa 1–126) did not bind to Bim, but it could bind Bcl-xL (Figure S1D), suggesting that the region in Beclin 1 sufficient for Bcl-xL binding is different from that for Bim. To avoid the cell death caused by wild-type Bim overexpression, we used *Bax/Bak* double-knockout (DKO) mouse embryonic fibroblasts (MEFs), where mitochondrially mediated cell-death pathways are deficient (Wei et al., 2001). We confirmed that the Bim-Beclin 1 interaction is independent of Bcl-2/Bcl-xL overexpression by introducing HA-tagged wild-type Bim and Flag-tagged Beclin 1 into *Bax/Bak* DKO MEFs and immunoprecipitating either Beclin 1 with anti-Flag (Figure 1B) or Bim with anti-HA (Figure 1C). We also confirmed the endogenous Bim-Beclin 1 interaction in HeLa cells (Figure 1D).

To avoid Bim's strong killing capability, we generated a Bim mutant that cannot bind to Bcl-2/Bcl-xL (thus loses toxicity) but can bind Beclin 1. Since L152 and F159 in Bim's BH3 domain are conserved in different BH3-only proteins (Figure S1E) and are thought to be crucial for apoptosis induction, we generated Bim L152E F159E (BimEE) and showed that it did not bind to Bcl-xL, like wild-type Bim (Figure 1E), or have wild-type Bim toxicity (Figures 1F–1H). However, BimEE could still bind to Beclin 1 (Figure 1I). The Bim BH3 domain was not required for its interaction with Beclin 1 (Figure S1F). Although BimEE bound to Beclin 1, death-deficient Noxa, Noxa 3E (Chen et al., 2005) did not bind to Beclin 1 (Figure S1G). (We noted that untagged Bim expression always yielded lower levels of BimEL and greater amounts of BimL compared to HA-tagged Bim). The Bim-Beclin 1 interaction appeared to be stronger than the Bcl-2-Beclin 1 and Bcl-xL-Beclin 1 interactions (Figure S1H). Interestingly, BimEL-EE and BimL-EE bound similarly to Beclin 1, while the shortest isoform of this Bim death-deficient mutant, BimS-EE, bound much less efficiently, only retaining about 5% of the BimEL-EE-Beclin-1 binding (Figure 1J), suggesting that the region lost between BimS and BimL is important for the interaction.

### Autophagosome Formation in Bim-Depleted and -Overexpressing Cells

**Figure 2:**
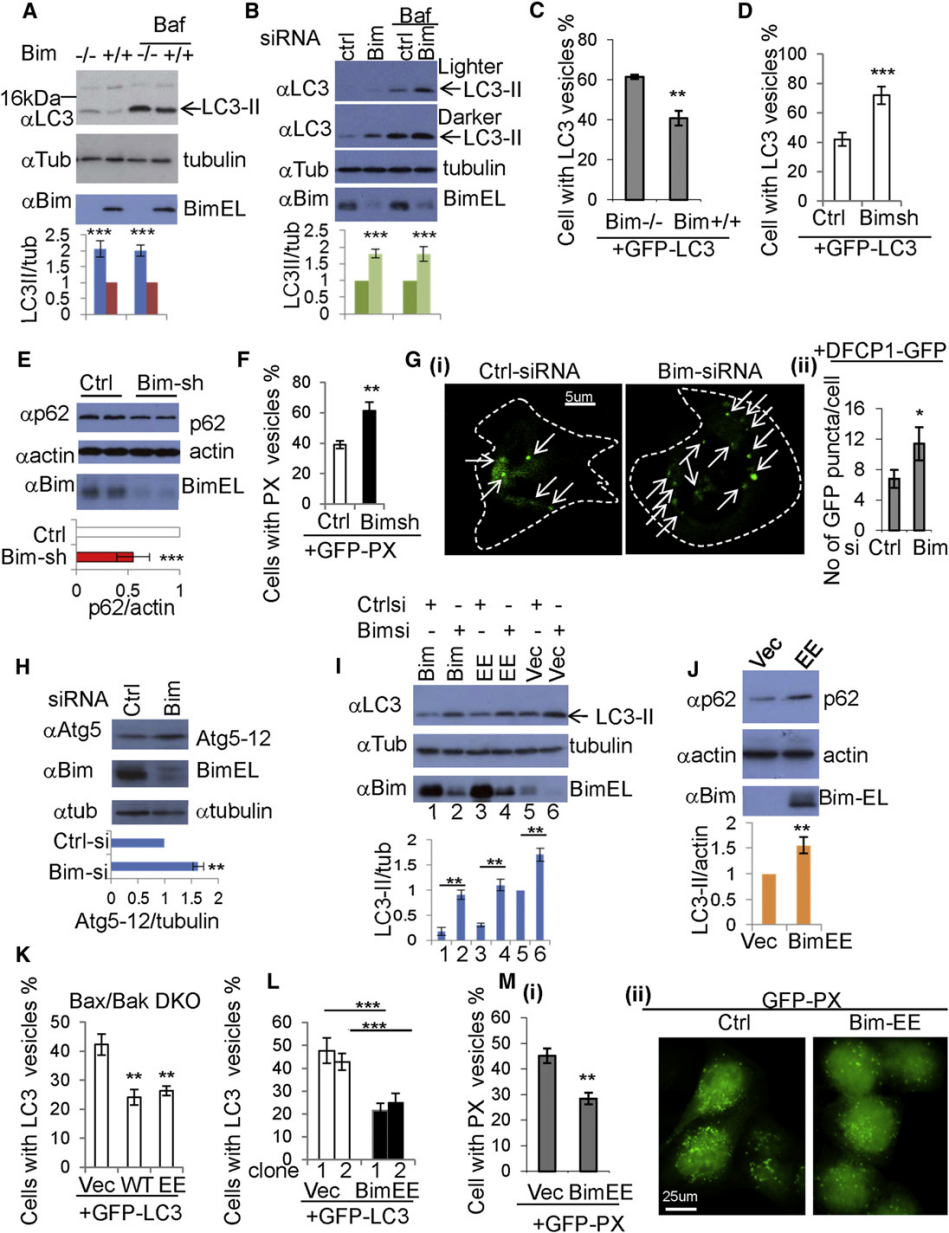
Autophagosome Levels in Bim-Depleted and -Overexpressing Cells (A) *Bim^−/−^* and *Bim^+/+^* MEFs were cultured in 6-well dishes. Cells were treated with vehicle or Bafilomycin A1 (Baf) for 4 hr. Blots were probed with the indicated antibodies. LC3-II/tubulin in wild-type control MEFs is set as 1. The relative value of LC3-II/tubulin in Bim-knockout MEFs is shown (n = 5). Data are mean ± SD. ^∗∗∗^p < 0.001. (B) HeLa cells were transfected with control siRNA and Bim siRNA. After 48 hr, cells were treated with vehicle or Baf for 4 hr. Blots were probed with the indicated antibodies and analyzed as described in (A). ^∗∗∗^p < 0.001. (C) *Bim^−/−^* and *Bim^+/+^* MEFs were transfected with GFP-LC3. The percentages of cells with GFP-LC3 vesicles were assessed. Data are shown as mean ± SD. ^∗∗^p < 0.01. (D) HeLa cells were transfected with control plasmid (pMKO)/GFP-LC3 or pMKO-BimshRNA/GFP-LC3 (to ensure that GFP-LC3-positive cells also contain BimshRNA or control plasmid, the ratio of pMKO or pMKO-BimshRNA/GFP-LC3 is 3:1). The percentages of cells with GFP-LC3 vesicles were assessed. Data are shown as mean ± SD. ^∗∗∗^p < 0.001. (E) HeLa cells were transfected with control plasmid (pMKO) or pMKO-BimshRNA. Blots were probed with the indicated antibodies. Data are shown as mean ± SD. ^∗∗∗^p < 0.001. (F) HeLa cells were transfected with control plasmid (pMKO)/GFP-PX or pMKO-BimshRNA/GFP-PX (3:1). The percentages of cells with GFP-vesicles were assessed. Data are shown as mean ± SD. ^∗∗^p < 0.01. (G) DFCP1-GFP cells were transfected with control siRNA or Bim siRNA. The numbers of GFP puncta were assessed. Data are shown as mean ± SD.^∗^p < 0.05. Images represent DFCP1-GFP vesicles in cells transfected with control siRNA and Bim siRNA. Arrows mark DFCP1-GFP puncta. (H) HeLa cells were treated with control siRNA or Bim siRNA. Blots were probed with the indicated antibodies. Data are shown as mean ± SD. ^∗∗^p < 0.01. (I) Bim(EL)/control siRNA, Bim(EL)/Bim siRNA, Bim(EL)EE/control siRNA, Bim(EL)EE/Bim siRNA, vector/control siRNA, and vector/Bim siRNA were transfected into HeLa cells. Blots were probed with the indicated antibodies. Data are shown as mean ± SD. ^∗∗^p < 0.01. (J) HeLa cells were transfected with control plasmid or Bim(EL)EE. Blots were probed with the indicated antibodies. Data are shown as mean ± SD. ^∗∗^p < 0.01. (K) *Bax/Bak* DKO MEFs were transfected with control plasmid /GFP-LC3, wild-type Bim(EL)/GFP-LC3 (3:1), or Bim (EL) EE/GFP-LC3. The percentages of cells with GFP-LC3 vesicles were assessed. Data are shown as mean ± SD. ^∗∗^p < 0.01. (L) Two independent control vector (pcDNA3) or Bim(EL)EE stably expressing HeLa cell clones were transfected with GFP-LC3 respectively. The percentages of cells with GFP-LC3 vesicles were assessed. Data are shown as mean ± SD. ^∗∗∗^p < 0.001. (M) HeLa cells were transfected with control plasmid/GFP-PX or Bim(EL)EE/GFP-PX (3:1). The percentages of cells with GFP-vesicles were assessed. Data are shown as mean ± SD. ^∗∗^p < 0.01. Fluorescent microscope images show GFP-PX vesicles. See also Figure S2.

We then assessed whether Bim regulated autophagy, since it binds Beclin 1. Autophagosome numbers correlate with the levels of the autophagosome-associated protein LC3-II (as a function of actin/tubulin) or the numbers of LC3-positive vesicles (Klionsky et al., 2008). We observed higher LC3-II levels in *Bim*-null MEFs than in wild-type MEFs (Figure 2A). Compared to wild-type MEFs, LC3-II levels were also increased in *Bim*-null MEFs treated with Bafilomycin A1, which inhibits degradation of LC3-II (Figure 2A), suggesting that *Bim*-null MEFs have higher rates of LC3-II formation. LC3-II levels were also increased by Bim small interfering RNA (siRNA) (Figures 2B and S2A) or Bim short hairpin RNA (shRNA) knockdown (Figure S2B), in the presence or absence of Bafilomycin A1, in HeLa cells, suggesting that endogenous Bim inhibits autophagosome formation. Likewise, the percentages of cells with GFP-LC3 vesicles were increased in *Bim*-null MEFs, compared to wild-type MEFs (Figure 2C), and in cells in which we knocked down Bim with shRNA (Figures 2D and S2C), compared to control cells.

Consistent with our data suggesting that Bim negatively regulates autophagy, we observed reduced levels of the autophagy substrate p62 (Bjørkøy et al., 2005) in cells in which we knocked down Bim with shRNA (Figure 2E). This effect appeared to be lysosome (autophagy)-dependent, since it was abrogated by Bafilomycin A1 (Figure S2D), and *p62* messenger RNA (mRNA) levels remained unchanged in Bim knockdown cells (Figure S2E). Autophagy regulates the proportion of cells with mutant huntingtin (htt) aggregates by regulating mutant htt clearance, and the percentage of cells with aggregates correlates inversely with autophagic activity (Luo and Rubinsztein, 2010; Ravikumar et al., 2004). The proportion of cells with mutant htt aggregates was reduced in cells transfected with Bim-shRNA (Figure S2F). Bim knockdown did not affect transferrin uptake and recycling, suggesting that the effects of Bim on autophagy are independent of the membrane trafficking pathways, like clathrin-dependent endocytosis, that regulate these processes (Ravikumar et al., 2010) (Figure S2G, panels i and ii).

Beclin 1 positively regulates the activity of the class III PI-3 kinase, Vps34, that generates phosphatidylinositol-3-phosphate (PtdIns3P), which is critical for autophagosome formation (Kihara et al., 2001). We used GFP-tagged PX domain of p40phox (GFP-PX) (Ellson et al., 2001) to quantify PtdIns3P-enriched structures and found these were increased by Bim knockdown (Figure 2F). Recently, DFCP1 has been shown to decorate PtdIns3P-enriched putative autophagosome precursors, called omegasomes (Axe et al., 2008), and these were also increased by Bim knockdown (Figure 2G). As Beclin 1/PtdIns3P may regulate Atg5-12 conjugation that is required for autophagosome formation (Luo and Rubinsztein, 2010; Ravikumar et al., 2008), we assayed this phenomenon and found it to be increased in Bim knockdown cells (Figure 2H). Furthermore, Bim knockdown increased the numbers of Atg12 vesicles (autophagosome precursors) (Figure S2H).

We then tested and confirmed that BimEE or Bim overexpression reversed the effect of Bim knockdown on LC3-II levels (Figure 2I). Bim knockdown increased LC3-II levels (lanes 5 and 6), while Bim (lanes 1 and 2) or BimEE (lanes 3 and 4) overexpression decreased both LC3-II levels in control siRNA- or Bim siRNA-transfected cells (Figure 2I). Also, BimEE overexpression increased p62 levels (Figure 2J). Wild-type Bim or BimEE overexpression decreased the percentage of cells with GFP-LC3 vesicles in Bax/Bak DKO MEFs (Figure 2K), and BimEE expression had similar effects in stably expressing HeLa cells (Figure 2L). BimEL-EE and BimL-EE significantly reduced autophagosome numbers, whereas BimS-EE, which binds Beclin 1 much more weakly (Figure 1J), did not affect GFP-LC3 vesicle numbers (Figure S2I). BimEE enhanced the percentage of mutant htt aggregate-positive cells in cells (Figure S2J). Consistent with these findings, overexpressed BimEE decreased GFP-PX vesicle numbers (Figure 2M, panels i and ii). These data reveal that Bim inhibits autophagy independently of its proapoptotic activity and this inhibition correlates with its ability to bind Beclin 1.

### Enhanced Autophagosome Formation in *Bim*-Knockout Mice

**Figure 3:**
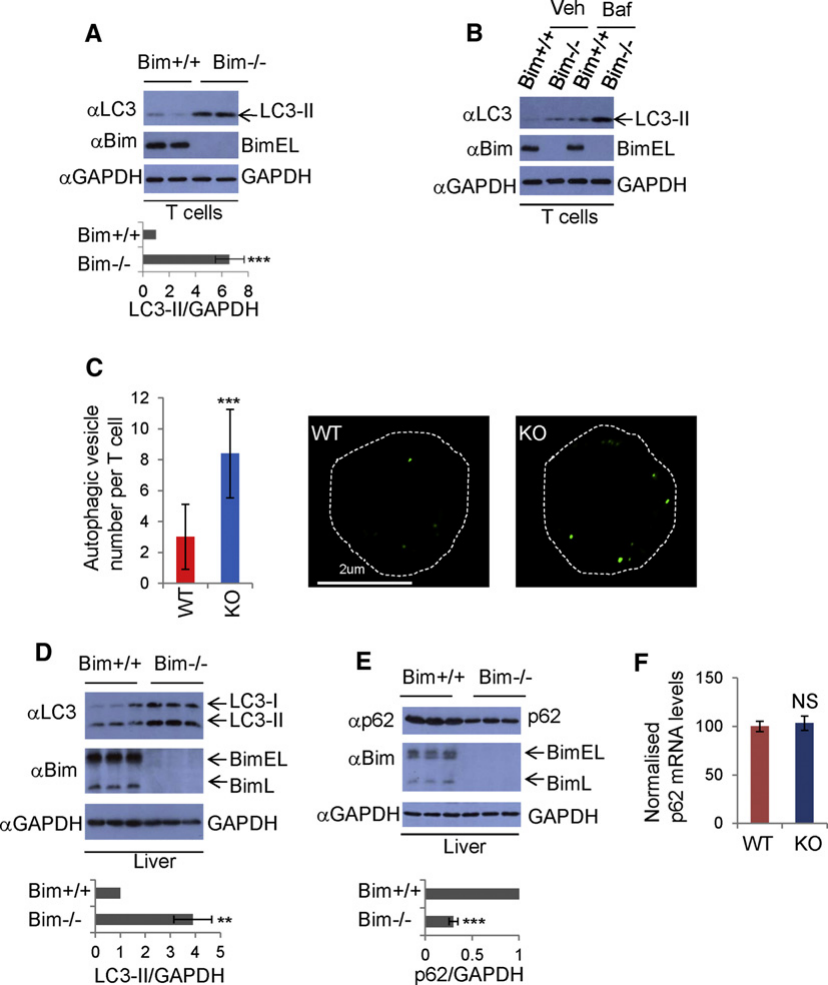
Autophagy Is Increased in *Bim*-Knockout Mice (A) Lysates from spleen-derived T cells of wild-type (*Bim^+/+^*) and *Bim*-knockout (*Bim^−/−^*) mice were immunoblotted and probed with indicated antibodies. Statistics were from three knockout and control mice. Data are shown as mean ± SD. ^∗∗∗^p < 0.001. (B) T cells from *Bim^+/+^* and *Bim^−/−^* mice were cultured with/without Baf for 4 hr preceding harvest, immunoblotted, and probed with the indicated antibodies. (C) Spleen-derived T cells from *Bim^+/+^* or *Bim^−/−^* mice were stained with rabbit anti-LC3 antibody. Representative confocal images and quantification from triplicates (n = 100–110) are shown. Data are shown as mean ± SD. ^∗∗∗^p < 0.0001. (D) *Bim^+/+^* and *Bim^−/−^* mouse liver lysates were immunoblotted and probed with the indicated antibodies (n = 3). Data are shown as mean ± SD. ^∗∗^p < 0.01. (E) *Bim^+/+^* and *Bim^−/−^* mouse liver lysates were immunoblotted and probed with the indicated antibodies (n = 3). Data are shown as mean ± SD. ^∗∗∗^p < 0.001. (F) RNA from *Bim* wild-type or *Bim*-knockout mouse livers was analyzed by qRT-PCR for *p62/beta-actin* mRNA. The mean ± SD from three mice per group is shown. NS, not significant. See also Figure S3.

We examined the effect of Bim on autophagosome formation in vivo and found markedly increased LC3-II levels in *Bim*-knockout mouse spleen cells compared with those in control mouse spleen cells (Figure S3A). We observed more LC3-II in *Bim*-knockout spleen cells, either in the presence or absence of Bafilomycin A1 (Figure S3B), further suggesting higher levels of autophagosome synthesis in *Bim*-knockout mice. Likewise, autophagosome numbers were elevated in *Bim*-knockout spleen cells (Figure S3C). Considering that spleen cells are composed of different cell types such as B cells and T cells, we then isolated T cells from the spleen cells and we observed significant increases of LC3-II and LC3 vesicles in T cells of *Bim*-knockout mice (Figures 3A–3C). We also found more LC3-II (Figure 3D) and lower p62 levels in *Bim*-knockout mouse livers compared to controls (Figure 3E). We confirmed that *p62* mRNA levels did not change in *Bim*-knockout mice (Figure 3F).

### The Effects of Bim on Autophagy Is Beclin 1 Dependent

We then tested whether the effects of Bim on autophagy were dependent on Beclin 1. Since aa 149–243 in Beclin 1, which is crucial for Bim binding (Figures S4A and S4B), also binds UVRAG and Atg14 (Sun et al., 2008), we could not use Bim binding-deficient Beclin 1 to test the possibility that the effect of Bim on autophagy is dependent on Beclin 1, since Bim binding-deficient Beclin 1 is also autophagy deficient (Figure S4C).

**Figure 4:**
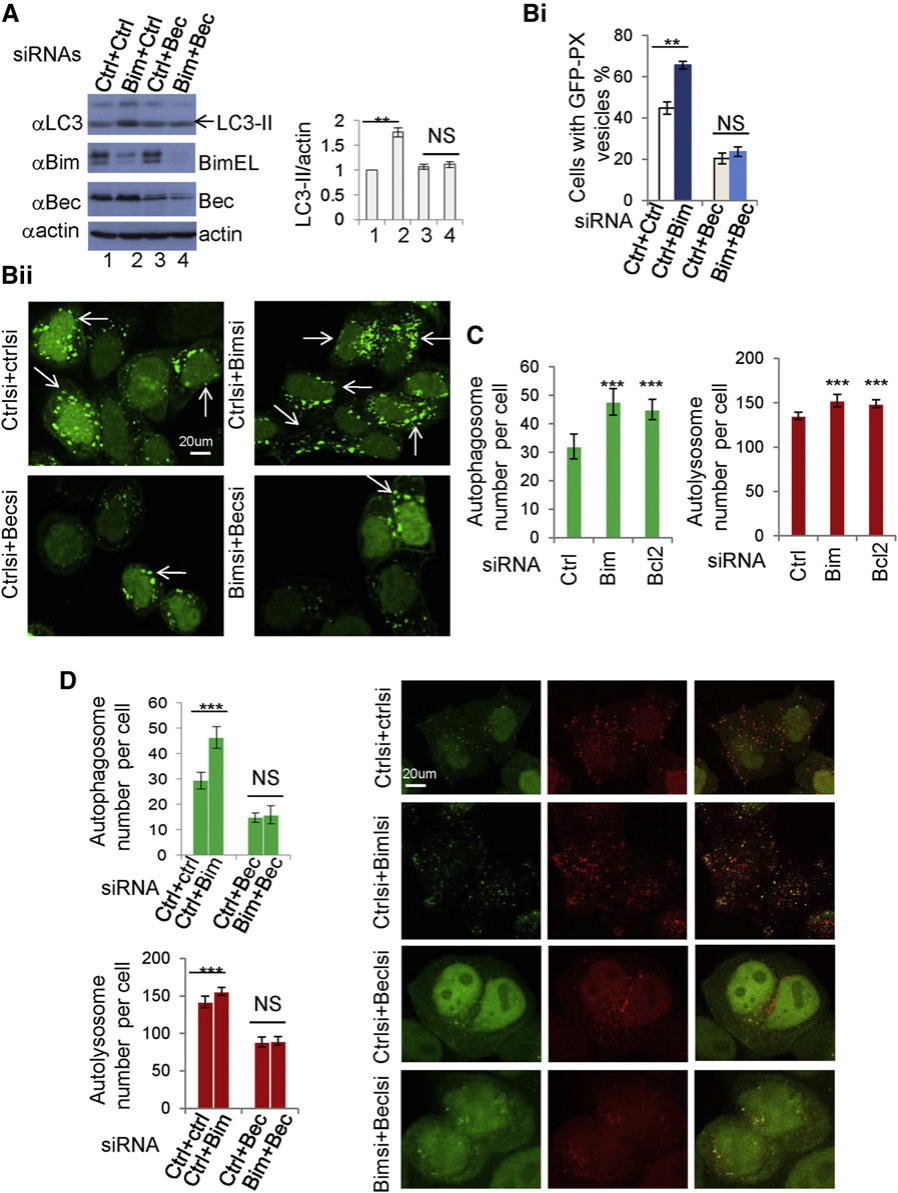
The Effect of Bim on Autophagy Is Beclin 1 Dependent (A) HeLa cells were treated with control siRNA, Bim siRNA + control siRNA, control siRNA + Beclin 1 siRNA, or Bim siRNA + Beclin 1 siRNA. Blots were probed as indicated. Data are shown as mean ± SD. ^∗∗^p < 0.01. (B) HeLa cells were treated with control siRNA, Bim siRNA + control siRNA, control siRNA + Beclin 1 siRNA, or Bim siRNA + Beclin 1 siRNA. After 24 hr, cells were transfected with GFP-PX for 24 hr. GFP-PX vesicles were assessed with a confocal microscope. Data are shown as mean ± SD. ^∗∗^p < 0.01. Arrows label cells with increased number/size of GFP-PX vesicles. (C) GFP-mRFP-LC3 HeLa cells were treated with control, Bim siRNA, or Bcl-2 siRNA and then analyzed with Cellomics microscope. Data are shown as mean ± SD. ^∗∗∗^p < 0.001. (D) GFP-mRFP-LC3 stable HeLa cells were treated with control siRNA, Bim siRNA + control siRNA, control siRNA + Beclin 1 siRNA, or Bim siRNA + Beclin 1 siRNA and then analyzed with Cellomics microscope and matching confocal images are shown. Data are shown as mean ± SD. ^∗∗∗^p < 0.001. See also Figure S4.

Accordingly, we tested whether Bim influenced autophagy in Beclin 1-depleted cells, and we found that Bim knockdown failed to increase LC3-II levels when Beclin 1 was knocked down (Figure 4A), suggesting that the effect of Bim on LC3-II formation is Beclin 1 dependent. Likewise, Beclin 1 depletion abolished the ability of Bim siRNA to increase the number of PX vesicles (Figure 4B, panels i and ii, and Figure S4D).

Beclin 1 regulates both autophagosome synthesis and autophagosome maturation (Liang et al., 2008; Matsunaga et al., 2009; Zhong et al., 2009). GFP-mRFP-LC3 vesicle analysis allows us to monitor autophagosome synthesis and autophagosome maturation by labeling autophagosomes (green and red) and autolysosomes (red), since the low lysosomal pH quenches the GFP more quickly (Kimura et al., 2007). In cells where Bim or Bcl-2 was knocked down by siRNA, we observed a marked increase of autophagosome number and smaller but significant increase of autolysosome number (Figures 4C and S4E). We confirmed that both Bim and Bcl-2 knockdown increases LC3-II (Figure S4F). The smaller increase of autolysosomes caused by Bim or Bcl-2 knockdown may reflect the fact that LC3-II is degraded in lysosomes, and LC3-II in autolysosomes may have a shorter half-life than in autophagosomes (Figure 4C). Furthermore, we observed that the effect of Bim on both autophagosome and autolysosome numbers is Beclin 1 dependent, since the effect was disabled in cells where Beclin 1 was knocked down (Figures 4D and S4G).

### Starvation Disrupts the Beclin 1-Bim Interaction

**Figure 5:**
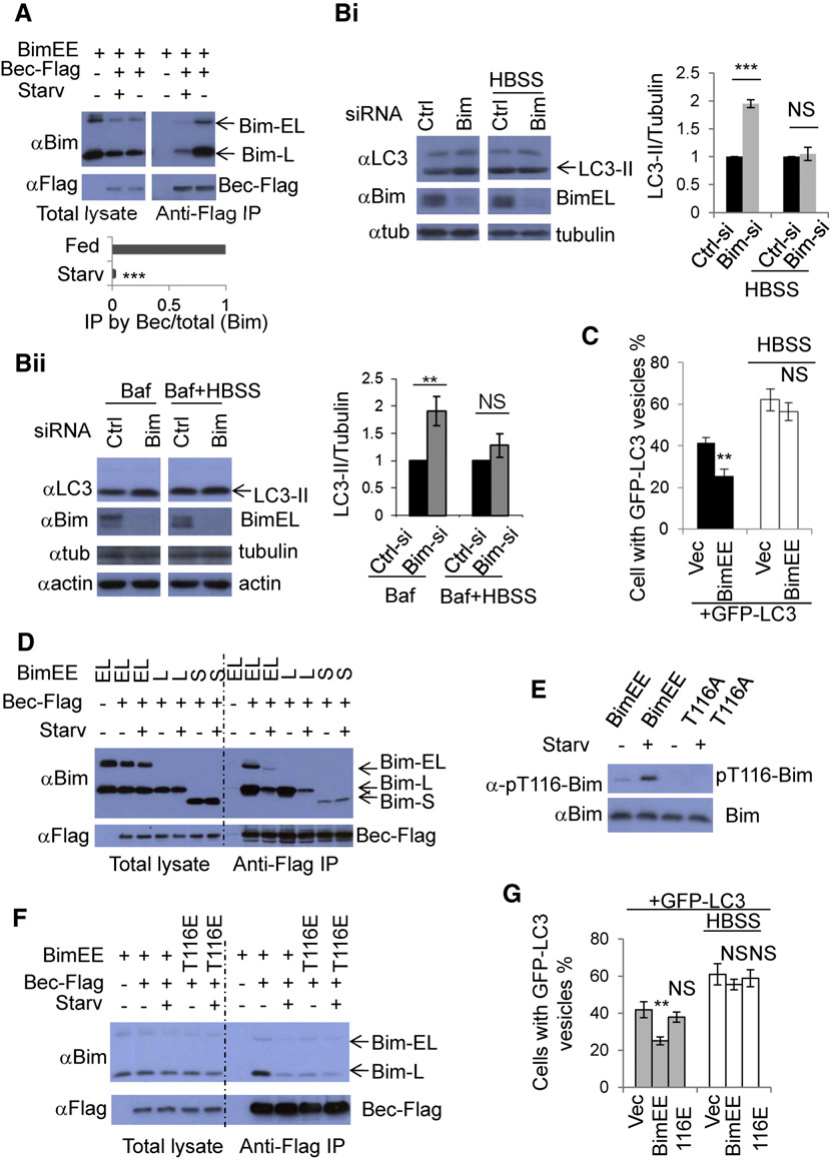
Starvation-Induced BimT116 Phosphorylation Disrupts the Bim-Beclin- 1 Interaction (A) Bim(EL)EE/empty vector (IP negative control) or Bim(EL)EE/Beclin 1-Flag (in duplicate) were transfected into HeLa cells. After 20 hr, one set of cells with Bim(EL)EE/Beclin 1-Flag were starved in HBSS for 2 hr. Anti-Flag antibody (M2) was used for immunoprecipitation, and blots were probed as indicated. Data are shown as mean ± SD. ^∗∗∗^p < 0.001. (B) Panel i: HeLa cells were treated with control siRNA or Bim siRNA. After 48 hr, one set of transfections were starved in HBSS for 2 hr. Blots were probed as indicated. LC3-II/tubulin ratio of siRNA-transfected cells is set as 1 (n = 3). Data are shown as mean ± SD. ^∗∗∗^p < 0.001; NS, not significant. Panel ii: HeLa cells were treated with control siRNA or Bim siRNA. After 48 hr, one set of transfections were treated with Baf for 2 hr; one set of transfections were starved and treated with Baf for 2 hr. Blots were probed as indicated and analyzed as in (Bi). ^∗∗^p < 0.01; NS, not significant. (C) HeLa cells were transfected with control plasmid /GFP-LC3 (3:1), or Bim(EL)EE/GFP-LC3 (3:1). After 20 hr, one set of transfected cells were starved in HBSS for 2 hr and then fixed. The percentages of cells with GFP-LC3 vesicles were assessed. Data are shown as mean ± SD. ^∗∗^p < 0.01. NS, not significant. (D) BimS-Beclin 1 binding is not sensitive to HBSS starvation. BimEL-EE (EL)/vector (IP negative control), BimEL-EE (EL)/Beclin 1-Flag (two replicates), BimL-EE (L)/Beclin 1-Flag (two replicates) or BimS-EE (S)/Beclin 1-Flag (two replicates) were transfected into HeLa cells. After 20 hr, one of EL-Beclin 1-Flag transfections, one of L-Beclin-Flag transfections and one of S-Beclin 1-Flag transfections were subjected to 2 hr HBSS starvation. Anti-Flag (M2) was used for immunoprecipitation. (E) HBSS starvation increases Bim T116 phosphorylation. Bim(EL)EE or BimEE(EL)-T116A were transfected into HeLa cells. After 20 hr, one set of Bim(EL)EE transfections and one of Bim(EL)EE-T116A were starved for 2 hr in HBSS. Blots were probed with the indicated antibodies. (F) Bim T116 phosphorylation disables Bim-Beclin 1 interaction. Bim(EL)EE /vector (IP negative control), Bim(EL)EE/Beclin 1-Flag (in duplicate), or Bim(EL)EE-T116E (phospho-mimic, designated as T116E here)/Beclin-Flag (in duplicate) were transfected into HeLa cells. After 20 hr, one set of Bim(EL)EE/Beclin 1-Flag transfections and one of Bim(EL)EE-T116E/Beclin 1-Flag transfections were starved for 2 hr in HBSS (Starv). Anti-Flag (M2) was used for immunoprecipitation. (G) HeLa cells were transfected with control plasmid /GFP-LC3, Bim(EL)-EE/GFP-LC3 (3:1), or Bim(EL)-EE T116E/GFP-LC3 (3:1). After 20 hr, one set of transfected cells were starved in HBSS for 2 hr and the cells were then fixed. The percentages of cells with GFP-LC3 vesicles were assessed. Data are shown as mean ± SD. ^∗∗^p < 0.01; NS, not significant. See also Figure S5.

Our data suggest that Bim interacts with Beclin 1 and that Bim inhibits autophagosome formation. Interestingly, Bim-Beclin 1 binding was dramatically weakened when cells underwent nutrient starvation (without serum and amino acids), which also induces autophagy (Levine and Klionsky, 2004; Schmelzle and Hall, 2000) (Figure 5A). Time-course experiments show that 30 min Hank's solution (HBSS) starvation started to affect the Bim-Beclin 1 interaction significantly and that the effect was dramatic after 2 hr (Figure S5A). We found that 1 hr full medium recovery allowed some restoration of the Bim-Beclin 1 interaction in cells previously starved for 2 hr (Figure S5B). Verapamil and calpastatin are two recently identified autophagy inducers (Williams et al., 2008). Unlike starvation, these two agents only modestly inhibited the Bim-Beclin 1 interaction (Figure S5C). We then tested whether Bim knockdown still induced LC3-II formation in starved cells, since the Bim-Beclin 1 interaction is vastly weakened in these conditions (Figures 5A and S5A). We observed that the differences in LC3-II formation in control siRNA- and Bim siRNA-treated cells were lessened in starvation conditions, compared to nonstarvation conditions, with or without Bafilomycin A1 treatment (Figure 5B, panels i and ii). Likewise, while Bim overexpression inhibits autophagosome formation in nutrient-replete conditions, this effect was abolished in starved cells (Figure 5C). Since BimEL and BimL bind Beclin 1 similarly, and the BimS-Beclin 1 interaction is much weaker (see Figure 1J), we examined whether BimS binding to Beclin 1 is modulated by HBSS starvation. Importantly, we observed that the weak interaction between BimS and Beclin 1 remained unchanged after HBSS starvation (Figure 5D). This result suggests that the sequence difference between Bim-L and Bim-S (aa 102–131) is responsible for regulating the Bim-Beclin 1 interaction in starvation conditions (Figure S5D). It is possible that the apparent decrease in Bim levels after HBSS starvation (Figure S5A) may also be an additional factor in autophagy regulation.

We speculated that phosphorylation or dephosphorylation may mediate the weakened Bim-Beclin 1 interaction in starved cells. We identified six potential phosphorylation sites within aa 102–131 of Bim (EL) that discriminates BimL and BimS (Figure S5D), of which S104, T116, and S118 are potential JNK phosphorylation sites (Lei and Davis, 2003). Mutations of single or multiple sites in this domain suggested that T114 and T116 could regulate Beclin 1 binding (Figures S5E–S5G). As JNK activity is enhanced during nutrient starvation (Geeraert et al., 2010; Wei et al., 2008) (see Figure S5H), and it can phosphorylate Bim T116 (equivalent to BimL T56 and mouse Bim T112) and disrupt the LC8-Bim interaction (Lei and Davis, 2003), we tested and confirmed that this site was phosphorylated in our starvation conditions (Figure 5E), that a phosphomimetic T116E mutation decreased Beclin 1 binding to a similar extent to that seen with starvation and that BimT116E-Beclin 1 binding appeared no longer sensitive to starvation (Figure 5F). We confirmed that BimT116E did not inhibit GFP-LC3 vesicle formation like wild-type Bim (Figure 5G). Thus, this phosphorylation may be a mechanism whereby starvation influences both the Bim-Beclin 1 interaction and autophagy.

### LC8 Promotes the Bim-Beclin 1 Interaction

**Figure 6:**
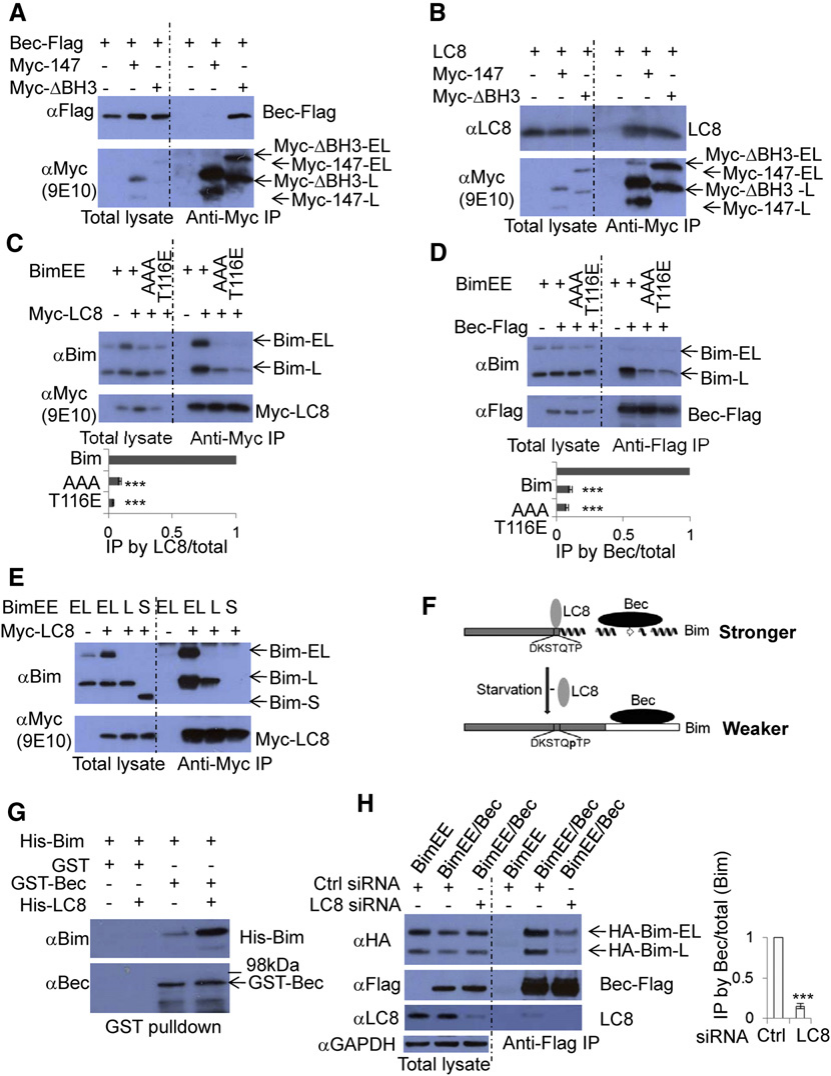
LC8 Promotes the Bim-Beclin 1 Interaction (A) Bim-147 does not bind to Beclin 1. Beclin 1-Flag/vector (IP negative control), Beclin 1-Flag/Myc-BimEL-1-147aa (Myc-147), or Beclin 1-Flag/Myc-BimEL-ΔBH3 (Myc-ΔBH3) were transfected into HeLa cells. Anti-Myc (Rabbit) was used for immunoprecipitation. (B) Bim-147 is competent for LC8 binding. LC8/vector (IP negative control), LC8/Myc-BimEL-1-147aa (Myc-147), or LC8/Myc-BimEL-ΔBH3 (Myc-ΔBH3) were transfected into HeLa cells. Anti-Myc (Rabbit) was used for immunoprecipitation. (C) Mutating LC8 binding consensus sites within Bim disrupts the Bim-LC8 interaction. Bim(EL)EE/vector (IP negative control), Bim(EL)EE/Myc-LC8, Bim(EL)EE-S109A, S113A, T114A (AAA)/ Myc-LC8, or Bim(EL)EE-T116E/ Myc-LC8 were transfected into HeLa cells. Anti-Myc (Rabbit) was used for immunoprecipitation. Data are shown as mean ± SD. ^∗∗∗^p < 0.001. (D) Mutating LC8 binding consensus sites within Bim disrupts the Bim-Beclin 1 interaction. Bim(EL)EE/vector (IP negative control), Bim(EL)EE/Beclin 1-Flag, Bim(EL)EE-S109A, S113A, T114A (AAA)/Beclin 1-Flag, or Bim(EL)EE-T116E/Beclin 1-Flag were transfected into HeLa cells. Anti-Flag (M2) was used for immunoprecipitation. Data are shown as mean ± SD. ^∗∗∗^p < 0.001. (E) Bim-S does not bind to LC8. BimEL-EE (EL)/vector (IP negative control), BimEL-EE (EL)/Myc-LC8, BimL-EE (L)/Myc-LC8, or BimS-EE (S)/Myc-LC8 were transfected into HeLa cells. Anti-Myc (Rabbit) was used for immunoprecipitation. (F) Model of LC8 promoting Beclin 1-Bim interaction. In nutrients, LC8 binds the Bim consensus sequence (DKSTQTP). This enables a transition of the Bim C terminus from a disordered state to α helices and a β sheet (Barbar, 2008), which favors the Bim-Beclin 1 interaction. In starvation conditions, Bim is phosphorylated at T116 within the consensus sequence (DKSTQpTP) leading to its dissociation from LC8 (Lei and Davis, 2003). Bim returns to the disordered state, which binds to Beclin 1 less effectively. Grey boxes, higher degree of disorder; white box, lower degree of disorder (Barbar, 2008). (G) LC8 enhances Bim-Beclin 1 interaction in vitro. His-Bim, His-LC8 and GST-Beclin 1 were expressed in BL21(DE3) *E. coli* and purified. Two micrograms of His-Bim was combined with 2 μg GST without or with 1 μg His-LC8 (as controls); 2 μg His-Bim was combined with 2 μg GST-Beclin 1 without or with 1 μg His-LC8. The mixtures were incubated in buffer A for 3 hr. Glutathione beads were then used to pull down GST or GST-Beclin 1. The pulldown products were detected with anti-Bim and anti-Beclin 1. (H) LC8 siRNA knockdown reduces the Bim-Beclin 1 interaction. Control siRNA or LC8 siRNA were transfected into HeLa cells. After 48 hr, HA-Bim(EL)EE/vector (IP negative control) or HA-Bim(EL)EE/Beclin 1-Flag was transfected into control siRNA transfected HeLa cells; HA-Bim(EL)EE/Beclin 1-Flag was transfected into LC8 siRNA-transfected HeLa cells. Anti-Flag (M2) was used for immunoprecipitation. Note that in the presence of BimEE, endogenous LC8 was pulled down by Beclin 1 when cells were treated with control siRNA. Data are shown as mean ± SD. ^∗∗∗^p < 0.001. See also Figure S6.

Our observation that both the phosphomimetic (T116E) and nonphosphorylatable (T116A) Bim mutants had impaired Beclin 1 interactions (Figure S5G) suggested that T116 was in a binding site that was relevant and that either mutation would interfere with the Bim-Beclin 1 interaction. We noted that LC8 binds Bim at the consensus site KXTQTX (Barbar, 2008), where the latter T is position 116 (Figures S5D and S6A). We wondered why mutation/phosphorylation in the LC8-binding domain of Bim affected the Bim-Beclin 1 interaction. We considered it unlikely that Beclin 1 would compete with LC8 for binding to the same region on Bim, since when we immunoprecipitated Beclin 1, we pulled down both Bim and LC8, and the ability of Beclin 1 to interact with LC8 is dependent on forms of Bim that bind Beclin 1 (Figure S5F) (Figure S6B confirms previous data [Puthalakath et al., 1999] suggesting that endogenous Bim binds LC8.) Indeed, Bim residues 1–147 are not sufficient for Beclin 1 binding (Figure 6A), while they do bind LC8 (Figure 6B), suggesting that the region of Bim for Beclin 1 binding is different from that for LC8 binding (see Figure S6C). However, Bim mutants that bind LC8 weakly (Figure 6C), also bind Beclin 1 weakly (Figure 6D), and BimS that does not bind LC8 (Figure 6E), binds Beclin 1 weakly (Figure 1J) (see Figure S6D). Thus, we hypothesized that LC8 binding to Bim would enhance the ability of Bim to bind Beclin 1 (Figure 6F), which was fuelled by our observation that Beclin 1 could coimmunoprecipitate BimEE with endogenous LC8 (Figure S5F). In this model (Figure 6F), starvation-induced Bim phosphorylation at T116 disrupts the LC8 binding consensus site in Bim and results in the dissociation of Bim and LC8, leading to the dissociation of Bim and Beclin 1. This model (Figure 6F) was supported by our observation that recombinant Bim and Beclin 1 interacted but this interaction was enhanced by the presence of LC8 (Figure 6G and S6E). Likewise, in cells, LC8 knockdown weakened the Bim-Beclin 1 interaction (Figure 6H). This effect is likely due to LC8 altering the conformation of Bim, as has been previously proposed (Barbar, 2008), as opposed to LC8 additionally binding to Beclin 1 (Figures S6F and S6G). Interestingly, Ambra1 is also able to bind Beclin 1 and LC8 (Di Bartolomeo et al., 2010). We observed that Ambra1 is expressed in HeLa cells at very low levels compared to other cells (Figure S6H). The Bim-Beclin 1 interaction increased when Ambra1 was overexpressed, and we also observed that more LC8 was pulled down by Bim in Ambra1-overexpressing cells (Figure S6I).

### Bim Cooperates with LC8 to Constrain Beclin 1

**Figure 7:**
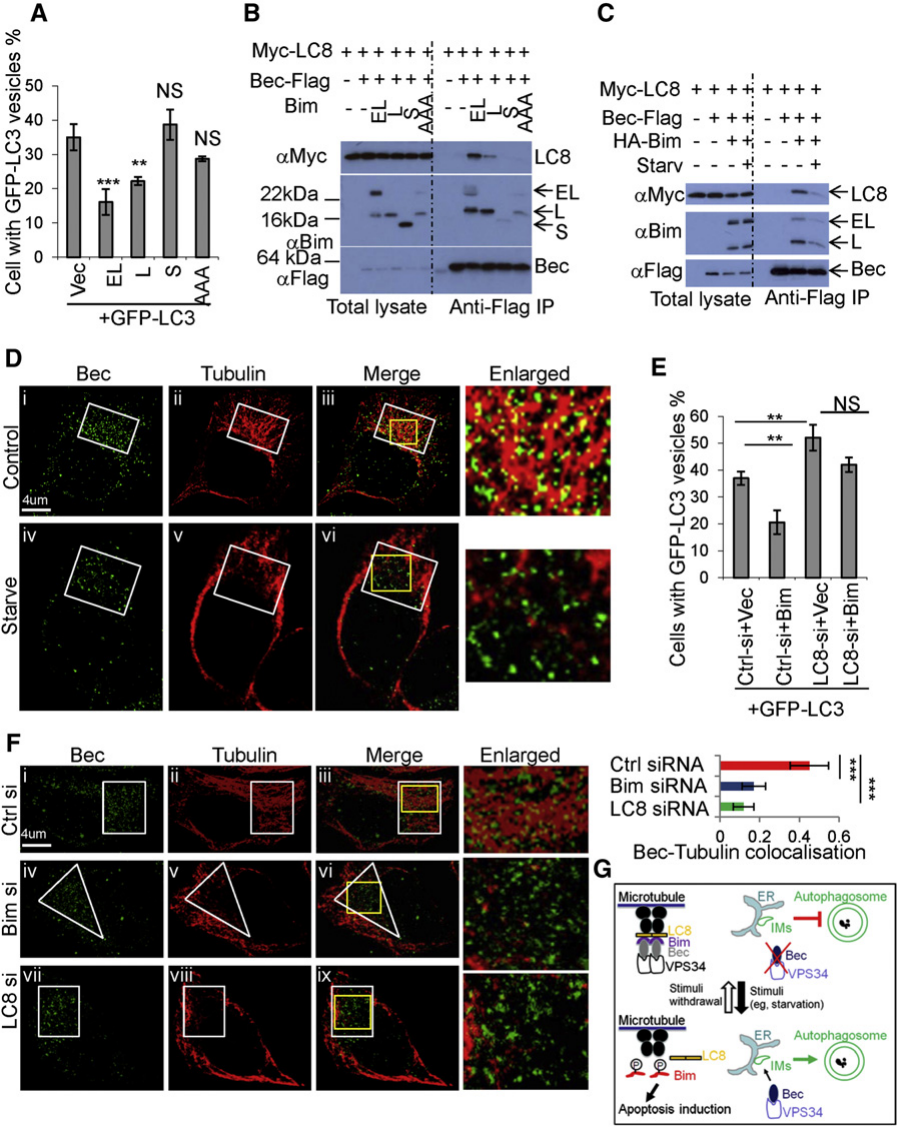
Bim Suppresses Autophagy by Mediating the LC8-Beclin 1 Interaction (A) HeLa cells were transfected with vector (vec) /GFP-LC3, BimEL-EE (EL)/GFP-LC3, BimL-EE (L)/GFP-LC3, BimS-EE (S)/GFP-LC3, or BimEL-EE-AAA (AAA)/GFP-LC3 (3:1). The percentages of cells with GFP-LC3 vesicles were assessed. Data are shown as mean ± SD. ^∗∗∗^p < 0.001; ^∗∗^p < 0.01; NS, not significant. Note that BimEL-EE-S109A, S113A, T114A is designated as BimEL-EE-AAA. (B) Bim bridges the Beclin 1-LC8 interaction. Dynein light chain1 (Myc-LC8)/empty vectors (IP negative control), Myc-LC8/Beclin 1-Flag/empty vector, Myc-LC8/Beclin 1-Flag/BimEL-EE (EL), Myc-LC8/Beclin 1-Flag/BimL-EE (L), Myc-LC8/Beclin 1-Flag/BimS-EE (S), and Myc-LC8/Beclin 1-Flag/BimEL-EE-S109A, S113A, T114A (AAA) were transfected into HeLa cells. Anti-Flag (M2) was used for immunoprecipitation. (C) Starvation reduces the ability of Bim to bridge the Beclin 1-LC8 interaction. Myc-LC8/empty vectors (IP negative control), Myc-LC8/Beclin 1-Flag/empty vector, or Myc-LC8/Beclin 1-Flag/HA-BimEL-EE (HA-Bim) (two replicates) were transfected into HeLa cells. After 20 hr, one set of cells with Myc-LC8/Beclin 1-Flag/HA-HA-Bim was starved in HBSS for 2 hr. Anti-Flag (M2) antibody was used for immunoprecipitation. (D) HeLa cells were cultured with DMEM with 10% serum (Control) or starved (Starve) in HBSS for 2 hr. The fixed cells were stained with Beclin 1 and tubulin antibodies and analyzed by confocal microscopy. White boxes show the areas where Beclin 1 is enriched. Yellow boxes show enlarged areas. (E) HeLa cells were treated with control siRNA or LC8 siRNA. After 24 hr, cells were split. Vector/GFP-LC3 or Bim(EL)EE/GFP-LC3 (3:1) were transfected into the control siRNA-transfected or LC8 siRNA-transfected cells. The percentages of cells with GFP-LC3 vesicles were assessed. Data are shown as mean ± SD. ^∗∗^p < 0.01; NS, not significant. (F) HeLa cells were treated with control siRNA, Bim siRNA, or LC8 siRNA for 48 hr. Cells were then fixed in 37°C, 4% PFA for 10 min. Cells were stained with Beclin 1 and tubulin antibodies and analyzed by confocal microscopy. White boxes/triangle show areas where Beclin 1 is enriched. Yellow boxes show enlarged areas. Colocalizations were quantified from images in 12–15 cells with Volocity program (Colocalization coefficient Mx). Data are shown as mean ± SD. ^∗∗∗^p < 0.001. (G) Bim inhibits autophagy. In normal conditions, LC8 recruits Beclin 1 to the microtubule-based dynein motor complex via Bim, thereby inhibiting autophagosome formation (Bim possesses autophagy-inhibitory activity). Under stress conditions (e.g., nutrient starvation), Bim phosphorylation at T116 by JNK leads to its dissociation from LC8, and this dissociation triggers the dissociation of Bim-Beclin 1. The Beclin 1-Vps34 can then localize to isolation membranes (IMs) to enable autophagosome synthesis. Free Bim can induce apoptosis. See also Figure S7.

The Bim variants/isoforms (BimEL and BimL) that bind Beclin 1 efficiently are those that also bind LC8 (Figures 1J and 6C–6E; see summary in Figure S6D) and these forms of Bim also inhibit autophagy (Figure 7A), in contrast to forms of Bim that do not bind LC8 (Figure 7A). Thus, we tested whether the mechanism for autophagy inhibition was due to Beclin 1 being mislocalized from its site of autophagy action at the ER (Liang et al., 2001; Pattingre et al., 2005) to microtubules via a ternary interaction with LC8 mediated via Bim. Although there is no direct interaction between Beclin 1 and LC8 (Figures S6F, S6G, and 7B), the LC8 interaction with Beclin 1 was enabled by Bim (Figure 7B), and this was dissociated by starvation (Figure 7C). Consistent with our findings, starvation also reduced the Bim-LC8 interaction (Figure S7A). Immunostaining showed that the level of Beclin 1 colocalizing with microtubules in starved cells was markedly reduced, compared with that in control cells (Figures 7D and S7B). Likewise, LC8 siRNA abrogated the ability of Bim to inhibit autophagy (Figure 7E), and either Bim or LC8 siRNA decreased the association of Beclin 1 with microtubules, seen by immunostaining (Figures 7F and S7C) or fractionation assays (Figure S7D). Consistent with this model, the co-overexpression of LC8 and the form of Bim competent in binding Beclin 1 decreased ER localization of Bim (Figure S7E, panels i and ii).

Interestingly, Bim does not affect the Beclin 1-Vps34 interaction (Figure S7F). In the presence of Beclin 1, Bim was able to immunoprecipitate Vps34 (Figure S7G), although Bim did not bind Vps34 directly (Figure S7G). By contrast, Bim was unable to pull down UVRAG, in the presence of Beclin 1 (Figure S7H), presumably because the region in Beclin 1 for Bim binding overlaps that for UVRAG binding in Beclin 1 (Liang et al., 2006; Sun et al., 2008) and consequently Bim displaces the Beclin 1-UVRAG interaction.

Based on these results, we propose a model in which, depending on the conditions, Bim's activity can switch from being autophagy inhibitory to proapoptotic (Figure 7G). In normal conditions, LC8-bound, apoptosis-inactive Bim inhibits autophagosome formation by recruiting the Beclin 1 complex to microtubules. However, in stress conditions, Bim is freed from LC8 to become proapoptotic, and the Beclin 1 complex is released from Bim to become active (see also Figure 7G legend).

## Discussion

Phylogenetic analyses suggest that different BH3-only proteins may have acquired BH3 motifs by convergent evolution and may have biological functions in addition to apoptosis regulation (Aouacheria et al., 2005). Bcl-2 binds Beclin 1 and inhibits autophagy (Pattingre et al., 2005), and the proapoptotic BH3-only proteins, Bad and BNIP3, disrupt the interaction between Bcl-2 and Beclin 1 and promote autophagy (Maiuri et al., 2007; Zhang et al., 2008). In contrast, our data reveal that as a BH3-only protein, Beclin 1 can physically interact with Bim, another BH3-only protein, and not only antiapoptotic Bcl-2 proteins.

We found that LC8 promotes the Bim-Beclin 1 interaction. Nutrient starvation, an autophagy-inducing stimulus, disrupts the Bim-Beclin 1 interaction by dissociating LC8 and Bim, which is phosphorylated and activated (for apoptosis) by JNK (Hübner et al., 2008; Lei and Davis, 2003). LC8 is an essential component of microtubule-based motor dynein, which mediates processes including chromosome segregation and vesicle trafficking (Barbar, 2008). LC8 also interacts with proteins that regulate apoptosis (Puthalakath et al., 1999), viral pathogenesis (Tan et al., 2007), and enzyme activity (Liang et al., 1999). LC8 was proposed to promote dimerization of disordered proteins by driving conformational changes (Barbar, 2008). Our data support the concept that LC8 not only functions as a component of dynein light chain but also plays a crucial role in regulating conformations of partially disordered proteins.

Our data reveal that apoptosis-inactive LC8-associated Bim interacts with Beclin 1 to inhibit autophagy. It is likely that JNK regulates the Beclin 1-Bim interaction in starvation conditions, although other kinases may also contribute. We reason that the weakened interaction between Beclin 1 and Bim is physiologically relevant to the regulation of autophagy by the classical stimulus of nutrient depletion. While Beclin 1 has been recently suggested to be associated with LC8 via Ambra1 (Di Bartolomeo et al., 2010), this is likely to be a distinct and possibly parallel process, since our data suggest LC8-Bim-Beclin 1 complex formation and reveal that Bim knockdown is sufficient to reduce Beclin 1 association with microtubules and this is linked to autophagy induction in cells and in vivo.

Our data thus provide an apoptosis-independent function for Bim and suggest that its activity can be switched between apoptosis induction and autophagy inhibition, depending on the cellular conditions. It appears that only microtubule-associated, nonapoptotic Bim inhibits autophagy. In response to stress stimuli, Bim dissociates from the microtubule-based dynein complex, and the autophagy inhibition is lifted immediately due to the dissociation of Bim from Beclin 1. However, once free of microtubules, Bim is free to move to mitochondria and becomes proapoptotic (Figure 7G). Thus, our work may suggest how autophagy and apoptosis can be coregulated. Since autophagy inhibition compromises cell survival (Levine and Kroemer, 2008), Bim is likely to have cytotoxic effects by inhibiting autophagy, even when it is bound to microtubules and is sequestered from its direct proapoptotic location at mitochondria.

## Experimental Procedures

Complete methods can be found in the Supplemental Experimental Procedures.

### Annexin V Staining

Transfected cells were trypsinized and washed twice with cold PBS, then resuspended in 1× binding buffer (10 mM HEPES [pH 7.4], 140 mM NaCl, 2.5 mM CaCl_2_) at 1 × 10^6^ cells/ml. We transferred 100 μl of cells to a FACS tube, added 5 μl Annexin V-APC (BD Pharmingen) and incubated for 15 min at room temperature. We added 400 μl 1× binding buffer into each tube and analyzed by flow cytometry.

### Cell Viability Assay

Cell survival was determined with the Cell Titer-Glo Luminescent Cell Viability Assay kit (Promega) to measure ATP levels, according to the manufacturer's instructions. Briefly, 100 μl Cell Titer-Glo reagent was added to the culture medium. Cells were placed on a shaker for 5 min and then incubated at room temperature for 10 min. The SPECTRA Max M5 reader was used to read luminescence.

### Endocytosis Assay

Endocytosis and recycling assays of transferrin were performed as previously described (Peden et al., 2004; Puri, 2009). In brief, for the uptake assay, cells were trypsinized, washed with PBS, and then incubated with 20 μg/ml transferrin-Alexa Fluo 647 (Invitrogen) at 37°C for various time intervals. Cells were fixed with 4% PFA, and FACS was used for the analysis. For the recycling assay, cells were incubated with 20 μg/ml transferrin-Alexa Fluo647 at 37°C for 30 min. After the incubation, cells were incubated with 200 μg/ml unlabelled transferrin at 37°C for various time intervals. Cells were then fixed with 4% PFA and FACS analysis was performed.

### GFP-mRFP-LC3 Assay

HeLa cells stably expressing GFP-RFP-LC3 were transfected with the indicated siRNA. After 48 hr, cells were split into 24 wells (two rows) of a 96-well plate. Meanwhile, cells were seeded in a well of 6-well plate for immunoblotting to check knockdown efficiency, and a well of 6-well plate was prepared for confocal imaging. After further 24 hr, cells were fixed in 2% PFA for 5 min. Cellomics (Arrayscan VTI) was used to score green and red vesicles. Green vesicles are considered to be autophagosomes, and red vesicles are considered to be both autophagosomes and autolysosomes. The number of autolysosomes was achieved by subtraction of the number of green vesicles from that of the red vesicles.

### Microtubule Fractionation

Microtubules were isolated according to the methods described by Avila et al. (2008). In brief, HeLa cells treated with control, Bim, LC8 siRNAs were lysed with Triton X-100, and the lysates were cleared by ultracentrifugation for 40 min at 100,000 g, 4°C. The cleared lysates were treated with taxol (20 μM) and GTP (1 mM). The lysates were loaded on a 1 ml 20% sucrose cushion and subjected to ultracentrifugation 30 min at 180,000 g, 25°C. The pellets (microtubules) were resuspended in 100 μl buffer I (0.1 M MES [pH 6.7], 2 mM EGTA, 1 mM MgCl_2_).

### Estimation of Aggregates

To ensure that cells with HA-74Q also contain study plasmids, we used a 3:1 ratio of study plasmid:HA-74Q. Approximately 200 transfected cells were assessed in multiple random visual fields per slide. All coverslips were scored with the observer blinded to the identity of the slides. Cells were analyzed with a fluorescent microscope (Eclipse E600, Nikon, Japan), as previously described (Luo and Rubinsztein, 2010). The figures show data from representative experiments in triplicate. Cells were counted as aggregate-positive if one or several aggregates were visible within a cell.

### ER Tracker Staining and Live Images

ER tracker red (1 μM) was added to HeLa cells in serum-free DMSO for 15 min and washed once with DMSO. Live images were taken under a LSM510 confocal microscope.

### Analysis of Autophagosomes/Vesicles

The percentage of cells with autophagosome/vesicles was assessed as previously described (Pattingre et al., 2005). In experiments requiring a precise assessment of vesicle number, the number of vesicles per cell in GFP-positive cells was determined, as described previously (Luo and Rubinsztein, 2010). A Cellomics microscope was used for the GFP-mRFP-LC3 assay.

### Statistics

A t test was used, while p values for binary outcome data were determined by unconditional logistical regression analysis using the general loginear option of SPSS 9.1 (^∗∗∗^p < 0.001, ^∗∗^p < 0.01, ^∗^p < 0.05; NS, not significant). Data from three independent experiments were analyzed (unless otherwise stated).

## Supplemental Information

Document S1. Supplemental Experimental Procedures and Figures S1–S7
