# Supplementary material for: Bim Inhibits Autophagy by Recruiting Beclin 1 to Microtubules
Source: Mol Cell. 2012 Aug 10;47(3-8):359–70. doi: 10.1016/j.molcel.2012.05.040 (PMC3419265; doi:10.1016/j.molcel.2012.05.040)
Supplement: Document S1. Supplemental Experimental Procedures and Figures S1–S7 [file mmc1.pdf]

Molecular Cell, *Volume 47*

Supplemental Information

Bim Inhibits Autophagy

by Recruiting Beclin 1 to Microtubules

Shouqing Luo, Moises Garcia-Arencibia, Rui Zhao, Claudia Puri, Pearl P.C. Toh, Oana Sadiq, and David C. Rubinsztein

SUPPLEMENTAL EXPERIMENTAL PROCEDURES

Antibodies and reagents

Rabbit polyclonal antibodies: anti-Beclin 1 (C-terminus) (1:1,000) (Sigma); anti-Beclin 1 (N-terminus) (1:1,000) (Sigma); anti-Bim (1:1,000) (Cell Signaling); anti-Bcl-xL (1:2,000) (BD); anti-LC3 (1:10,000) (Novus Biologicals); anti-Flag (1:1,000) (Sigma); anti-GFP (1:1,000) (BD); actin (1:1,000) (Abcam); anti-Myc (1:1,000) (Sigma); anti-phospho-SAPK/JNK (Thr183/Tyr185) (1:1,000) (Cell Signaling); Anti-Vps34 (1:1,000) (Zymed); Anti-UVRAG (1:1,000) (Abcam); anti-Atg5 (1:1,000) (Sigma); anti-Atg12 (1:1,000) (Cell Signaling); anti-Ambra1 (1:500) (AbD Serotec); anti-PARP p85 (1:1,000) (Promega); anti-Bcl-2 (1:300) (Santa Cruz). Rat monoclonal antibody: anti-Bim (1:1,000) (Enzo). Mouse monoclonal antibodies: Beclin 1 (Sigma) (1:1,000), anti-Flag (M2) (1:1,000), anti-tubulin (1:5,000) (Sigma), anti-Myc (9E10) (1:1,000) (Sigma); anti-HA (1:1,000) (Covance); anti-LC8 (1:10,000) (Novus Biologicals); anti-GAPDH (1:5,000) (Ambion); anti-p62 (1:1,000) (BD); anti-JNK (1:1,000) (Santa Cruz). Anti-Flag M2-agarose affinity gel (Sigma). Anti-pT116-Bim was kindly offered by Dr R. J. Davis (University of Massachusetts Medical Center, Worcester) (Hubner et al., 2008). Bafilomycin A1 (Baf) was purchased from Millipore (400 nM).

DNA construction

Bim and Beclin 1 point mutants were generated with the Stratagene Quikchange Mutagenesis kit. Bad cDNA was purchased from Cell Signaling and subcloned into pcDNA3. HA-Bim, HA-Bak, HA-Puma, HA-Noxa, HA-Noxa-3E were kindly provided by Dr David Huang (Chen et al., 2005). BimL, BimS mutants (BimL-EE, BimS-EE) corresponding to BimEL-L152E F159E (BimEE/BimEL-EE) were generated by 3-step PCRs. BimΔBH3 was generated by 3-step PCRs. Bcl-xL, Vps34, and Beclin 1 plasmids were described earlier (Luo and Rubinsztein, 2010). Beclin 1 deletion mutants were generated by PCR and cloned into pCMV-5a (Sigma) for Flag-tagged proteins. LC8 IMAGE clone was purchased from Source Biosciences (Cambridge, UK) and LC8 cDNA was subcloned into pCMV-6M for Myc-LC8. UVRAG cDNA from an IMAGE clone (Source Biosciences) was subcloned into pcDNA3. Bim was subcloned into pCMV-6M for Myc-tagged Bim. Bcl-2, Bcl-xL were subcloned into pEF-HA-hygro to obtain HA-Bcl-2, HA-Bcl-xL. All the DNA constructs were confirmed by DNA sequencing. Bim small hairpin RNA construct, pMKO-BimshRNA (#17235) (Schmelzle et al., 2007) and its control plasmid, pMKO (#8452) (Stewart et al., 2003) were obtained from Addgene (Boston, MA). GFP-LC3ΔG plasmid (#24988) (Tanida et al., 2008) was from Addgene (Boston, MA). Ambra1 cDNA was from Origene.

Cell culture
Bim knockout and wild-type mouse embryonic fibroblasts (MEFs) were kindly provided by Dr Simon Cook (Babraham Institute, Cambridge, UK) (Ewings et al., 2007). Bax/Bak double-knockout (DKO) MEFs were kindly offered by Dr Christoph Borner (University of Freiburg, Germany) (Schinzel et al., 2004). HeLa, Bax/Bak DKO MEFs and Bim wild-type or knockout MEFs were cultured with standard methods in Dulbecco's Modified Eagle's Medium (DMEM) supplemented with 10% FCS (Sigma). For starvation, cells were washed once with Hank's Balanced Salt Solution (Sigma) (HBSS) and cultured in HBSS for the indicated time. DFCP1-GFP-expressing HEK293 cells (kind gift from Nick Ktistakis, Babraham Institute, Cambridge UK) were cultured in DMEM (10% FCS) containing 800 ug/ml G418 (Winslow et al., 2010). GFP-mRFP-LC3 stably expressing HeLa cells were cultured in DMEM (10% FCS) containing 500 ug/ml G418.

DNA and siRNA transfection
HeLa cells were split 1 day prior to transfection to 50% confluence and left overnight in antibiotic-free DMEM containing 10% FBS. siRNAs: Bim siRNA-1 (mostly used) (5'-GACCGAGAAGGUAGACAAUUG-3'), Bim siRNA-2 (5'-CAGGGAGACGUGUGAUAUA-3') and control siRNA (5'-CGUACGCGGAAUACUUCGA-3') (control siRNA-1) (Cell Signaling); LC8 siRNA (5'-GGACAUGUCGGAAGAGAUG-3'), Beclin 1 siRNA (5'-GGUCUAAGACGUCCAACAA-3'), Bcl-2 siRNA (5'-CCGGGAGAUAGUGAUGAAG-3') and control siRNA (5'-UGGUUUACAUGUCGACUAA-3') (control siRNA-2) (Dharmacon). Non-targeting siRNA was the control siRNA. DNA constructs and siRNAs were transfected with Lipofectamine 2000 according to the manufacturer's instructions. TransIT-2020 (Mirus) was used for Bax/Bak MEFs transfection. HeLa cells were maintained in 10% FBS DMEM containing no antibiotics for 48 hours after transfection.

Immunoprecipitation
Immunoprecipitation (IP) was performed using Buffer A (20 mM Tris-HCl, pH 7.4, 2 mM $MgCl_2$, 150 mM NaCl, 5 mM NaF, 1 mM Na3VO4, 0.5% NP-40, protease inhibitor cocktail (Roche)). Cells were lysed in Buffer A for 20 min on ice, followed by centrifugation at 13,000 x g for 15 min. 500 ug-1 mg total protein were used as the starting material for IPs. An antibody (or anti-Flag M2-agarose affinity gel) was added to a final concentration of 5ug/ml and incubated for 2 hours to overnight at 4°C. IP products were directly boiled in Laemmli buffer and subjected to PVDF membrane transfer and western blot.

Immunocytochemistry
Immunostaining was performed as previously described (Luo et al., 2005). After two washes with phosphate-buffered saline (PBS), cells were fixed with 4% paraformadehyde for 10 min. The fixed cells were washed three times in PBS, then permeablized with 0.5% Triton in PBS for 10 min. Cells were blocked in blocking buffer (1% BSA, 1% heat inactivated goat serum in PBS) for 30 min at room temperature. Primary antibodies were incubated with cells overnight at 4°C. The secondary antibody was incubated for 30 min after washing three times (10 min, each). Cells were washed three times (10 min, each) after incubation with secondary antibodies, then mounted with DAPI (3 ug/ml). Images were acquired on a LSM510 or LSM710 microscope.

RNA isolation and qRT–PCR analysis
Total RNA was isolated using Trizol reagent (Invitrogen) according to the manufacturer's instructions. For qRT–PCR analyses, equal amounts of RNA (0.5ug) were reverse-

transcribed by RT^2 first-strand kit (Qiagen), and then the resulting cDNA templates were subjected to qRT–PCR using the RT^2 SYBR Green Green Mastermix and RT^2 qPCR primers (Qiagen) with the 7900HT Fast Real-Time PCR system (Applied Biosystems). Beta-actin was used as a control to normalise the data. All primers were predesigned by Qiagen. Mouse p62 (Cat#: 330001 PPM28731E), Human p62 (Cat#: 330001 PPH02107A), Mouse beta-actin (Cat#: 330001 PPM02945A), Human beta-actin (Cat#: 330001 PPH0073E).

Production of GST-Beclin 1, His-Bim and His-LC8

Beclin 1 was subcloned into pGEX-6P-1 (Amersham) for recombinant GST-Beclin 1, and Bim or LC8 were cloned into pET-28a (Novagen) for His-tagged Bim or His-tagged LC8, respectively. The plasmids were then transformed into BL21 (DE3). A final concentration of 0.2 mM IPTG was added to culture media (LB) to induce recombinant protein expression.

GST-Beclin 1 was purified with glutathione-sepharose (GE Healthcare). Briefly, pellets were resuspended in PBS containing 1% Triton X-100 and protease inhibitor cocktail (Roche) and 0.1 mg/ml PMSF, then lysed by sonication. The lysates were subjected to 20,000 x g centrifugation, for 30 min. The supernatants were incubated with glutathione-sepharose beads for 2 hours. The glutathione-beads were washed with PBS and eluted with 20 mM of Glutathione in 50 mM Tris (pH8.0). Eluates were dialysed against binding buffer A (150 mM NaCl, 20 mM Tris, 2 mM $MgCl_2$, pH 7.4).

His-tagged proteins were purified with Ni^{2+}-charged His-tag affinity resins (Novagen) according to the manufacturer's instructions. The pellets were resuspended in binding buffer (0.5 M NaCl, 20 mM Tris, 5 mM imidazole, pH 7.9) containing protease inhibitor cocktail and 0.1 mg/ml PMSF, and sonicated. After centrifugation at 20,000 x g, 30 min, the supernatants were applied to His-affinity beads. The beads were washed with binding buffer and wash buffer (0.5 M NaCl, 20 mM Tris, 60 mM imidazole, pH 7.9) and eluted with elute buffer (0.5 M NaCl, 20 mM Tris, 1 M imidazole, pH 7.9). Eluates were dialysed against binding buffer A.

Mice

The strains of *Bim* knockout mice on a C57BL/6 background (stock number 004525) (Bouillet et al., 1999), and wild-type (WT) C57BL/6 mice (stock number 000664) were from the Jackson Laboratory. WT C57BL/6 mice were crossed with $Bim^{-/-}$ mice to generate $Bim^{+/-}$ mice. $Bim^{+/-}$ mice were crossed with $Bim^{+/-}$ mice to generate $Bim^{-/-}$ mice and $Bim^{+/+}$. Mice were genotyped by PCR using the following primers. Common: CATTCTCGTAAGTCCGAGTCT; wild-type: GTGCTAACTGAAACCAGATTAG; mutant: CTCAGTCCATTCATCAACAG (according to instructions from the Jackson Laboratory). All mice in this study were on a C57BL/6 genetic background. Littermate mice were used for each study. All animal work was performed according to UK Home Office legislation and with appropriate licences.

Spleen dissection

Briefly, spleens were collected in RPMI 1640 with 10% FBS. Spleen was broken with a plastic syringe plug. Spleen cells were collected by centrifugation. The cells were dissolved in 2 ml AKC buffer for 2 min. After centrifugation, AKC buffer was removed and cells were resuspended in RPMI 1640 with 10% FBS. Cell number was scored and $2x10^6$ cells were plated in per well of a poly-lysine-precoated 6-well plate or non-coated 6-well plate.

Mouse T cell Isolation

Mouse T cell isolation was performed with T cell enrichment columns from R&D according to the manufacturer's protocol after spleen cell preparation. Briefly, 2 ml suspension cells (~2

million spleen cells) were loaded onto a column. After 10 min incubation at room temperature, T cells were then eluted with 8 ml 1 x column wash buffer. The eluted T cells were cultured in RPMI-1640 and the autophagy assays were performed. Approximate 100 cells were assayed for autophagosome numbers.

Analysis of autophagosomes/vesicles

The percentage of cells with autophagosome/vesicles was assessed as previously described (Pattingre et al., 2005). Cells were counted as positive if GFP-LC3 vesicle number within a cell was greater than 50 (20 in *Bax/Bak* DKO MEFs as these cells have fewer LC3 vesicles) or if GFP-PX vesicle number within a cell was greater than 50. 200-400 cells were counted in multiple random visual fields per slide. The figures show data from experiments in triplicate, at least.

In experiments requiring a precise assessment of vesicle number, the number of vesicles per cell in GFP-positive cells was determined. Approximately 100 cells per sample were counted for triplicate samples, as described previously (Luo and Rubinsztein, 2010). All coverslips were scored with the observer blinded to the identity of the slides. A Cellomics microscope was used to quantify the GFP-mRFP-LC3 assays.

Quantification of autoradiographs

To quantify protein band density, the relevant specified bands were analysed using PhosphoImage software. The relative value was computed.

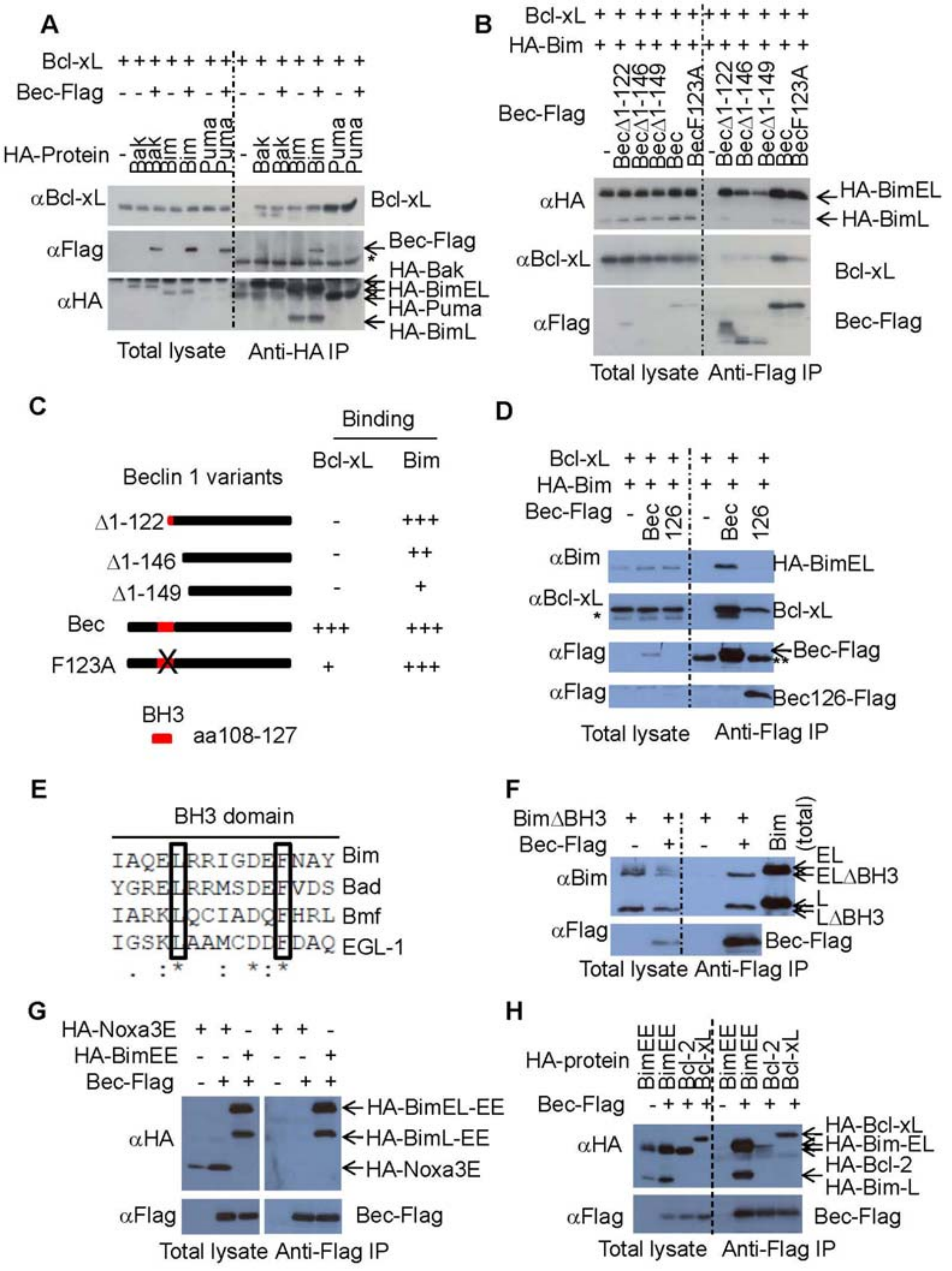

Figure S1. The Binding of Bim to Beclin 1 Is Distinct from the Bcl-xL-Beclin 1 Interaction and Death-Deficient Bim Retains Its Ability to Bind Beclin 1, Related to Figure 1

(A) Bcl-xL was cotransfected into HeLa cells with empty vector (IP negative control), HA-Bak, Beclin 1-Flag/HA-Bak, HA-Bim(EL), Beclin 1-Flag/HA-Bim(EL), HA-Puma, or Beclin 1-Flag/HA-Puma. After 20 hours, cells were lysed and anti-HA was used for

immunoprecipitation. Immunoprecipitates and total lysates were detected with anti-Bcl-xL, anti-Flag (M2) and anti-HA.

(B) HA-Bim(EL)/Bcl-xL were cotransfected into HeLa cells with empty vector (IP negative control), 1-122 aa-deleted Beclin 1–Flag–Beclin 1Δ1-122 (BecΔ1-122), Beclin 1Δ1-146-Flag (BecΔ1-146), Beclin 1Δ1-149-Flag (BecΔ1-149), Beclin 1-Flag (Bec), or Beclin 1-F123A-Flag (BecF123A). After 20 hours, cells were lysed and anti-Flag (M2) was used for immunoprecipitation. Immunoprecipitates and total lysates were detected with anti-HA, anti-Bcl-xL and anti-Flag (M2).

(C) Diagrams of Beclin 1 variants and their binding affinities to Bim and Bcl-xL (based on (B)). Beclin 1-BH3 domain (aa 108-127) is in red. +++: strong; ++: medium; +: weak.

(D) Bim(EL)/Bcl-xL was cotransfected into HeLa cells with empty vector (IP negative control), Beclin 1-Flag or Beclin 1 1-126aa-Flag (Bec-126-Flag). After 20 hours, cells were lysed and anti-Flag (M2) was used for immunoprecipitation. Immunoprecipitates and total lysates were detected with anti-Bcl-xL, anti-Bim and anti-Flag (M2). *: Bim band remaining after stripping, **: antibody heavy chain.

(E) BH3 domains from Bim, Bad, Bmf, EGL-1 were aligned. Boxed residues Leucine (L) and Phenylalanine (F), which are conserved across these BH3-only proteins and thought to be crucial for their roles in apoptosis induction, were mutated to Glutamate (E). The mutated Bim is designated as BimEE.

(F) Bim(EL)ΔBH3/empty vector (IP negative control), or Bim(EL)ΔBH3/Beclin 1-Flag were transfected into HeLa cells. After 20 hours, cells were lysed and the cellular lysates were subjected to anti-Flag (M2) immunoprecipitation. Immunoprecipitates and total lysates were resolved by SDS-PAGE and detected with anti-Bim and anti-Flag (Rabbit). Bim is shown in the upper panel.

(G) Death-defective mutant of Noxa (HA-Noxa3E)/empty vector (IP negative control), HA-Noxa3E/Beclin 1-Flag, or HA-Bim(EL)EE/Beclin 1-Flag were transfected into HeLa cells. After 20 hours, cells were lysed and anti-Flag (M2) was used for immunoprecipitation. Immunoprecipitates and total lysates were detected with anti-HA and anti-Flag (Rabbit).

(H) HA-BimEE(EL) (IP negative control), HA-Bim(EL)EE/Beclin 1-Flag, HA-Bcl-2/Beclin 1-Flag or HA-Bcl-xL/Beclin 1-Flag were transfected into HeLa cells. After 20 hours, cells were lysed and the cellular lysates were subjected to anti-Flag (M2) immunoprecipitation. Immunoprecipitates and total lysates were resolved by SDS-PAGE and probed with anti-HA and anti-Flag (Rabbit). Note that the level of HA-BimEE-L in total lysate is comparable to that of HA-Bcl-xL in total lysate.

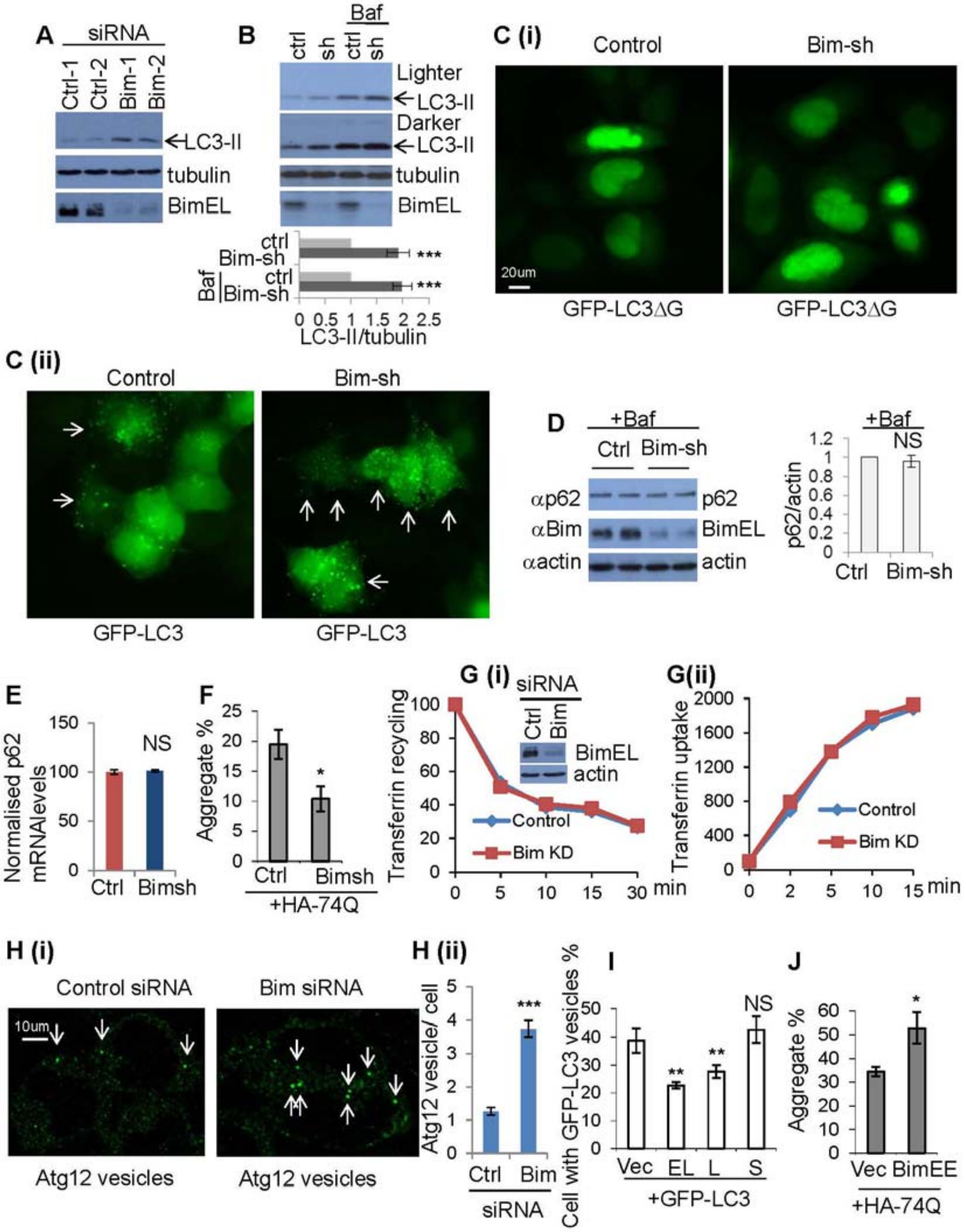

Figure S2. Autophagosome Formation in Bim-Depleted or Overexpressing Cells, Related to Figure 2

(A) HeLa cells were transfected with control siRNA-1, control siRNA-2 or Bim siRNA-1, Bim siRNA-2. After 48 hours cells were then harvested and subjected to SDS-PAGE. Blots were probed with anti-LC3, tubulin and Bim antibodies.

(B) HeLa cells were tranfected with control plasmid (pMKO) or pMKO-Bim-shRNA. After 48 hours, cells were treated with vehicle or Bafilomycin A1 (Baf) for 6 hours. Cell lysates

were subjected to SDS-PAGE and probed with anti-LC3, Bim and tubulin antibody. The ratio of LC3-II/tubulin in control-transfected HeLa cells is set as 1. The relative value of LC3-II/tubulin in Bim-shRNA-transfected cells is shown (n=3). Data are shown as mean±sd. **: P<0.01.

(C) Images show examples of GFP-LC3 vesicles in cells transfected with pMKO or pMKO-Bim-shRNA. The conjugation-deficient GFP-LC3ΔG was used as a control to show the authenticity of GFP-LC3 vesicles (Tanida et al., 2008). Arrows label the cells with increased number/size of GFP-LC3 vesicles.

(D) HeLa cells were transfected with control plasmid (pMKO) or pMKO-BimshRNA. After 48 hours, cells were treated with 100 nM Bafilomycin A1 (Baf) for 24 hours. Cells were then harvested and cell lysates were subjected to SDS-PAGE and blots were probed with anti-p62, Bim and actin antibodies. Quantifications are shown. Data are shown as mean±sd. NS: not significant.

(E) RNA was isolated from HeLa cells transfected with control vector or pMKO Bim-shRNA and subjected to analysis by qRT–PCR to detect the expression of *p62* mRNA. The mean ± SD of relative levels (normalized to beta-actin) from three independent experiments is shown. NS: not significant.

(F) HeLa cells were transfected with control plasmid (pMKO)/HA-httex1-74Q (3:1) or pMKO-Bim-shRNA/HA-httex1-74Q (3:1). After 24 hours, cells were fixed and stained with anti-HA antibody. The percentages of cells with aggregates were assessed. Data are shown as mean±sd.*: P<0.05.

(G) HeLa cells were transfected with control or Bim siRNA. After 48 hours, transferrin recycling (Y-axis: % of total transferrin internalised) (i) and transferrin uptake (Y-axis: % of plasma membrane amount) (ii) were performed.

(H) HeLa cells were treated with control or Bim siRNA. After 48 hours, cells were stained with Atg12 antibody. Images were taken under a confocal microscope. Atg12 vesicle number per cell was assessed. Data are shown as mean±sem. ***: P<0.001 (n=90-100).

(I) HeLa cells were transfected with control plasmid /GFP-LC3 (3:1), BimEL-EE (EL)/GFP-LC3 (3:1), BimL-EE (L)/GFP-LC3 (3:1) or BimS-EE (S)/GFP-LC3 (3:1). After 20 hours, cells were fixed. The percentages of cells with GFP-LC3 vesicles were assessed. Data are shown as mean±sd. **: P<0.01; NS: not significant.

(J) HeLa cells were transfected with control plasmid vector/HA-httex1-74Q, or Bim(EL)EE/ HA-httex1-74Q (3:1). After 48 hours, cells were fixed and stained with anti-HA antibody. Data are shown as mean±sd.*: P<0.05.

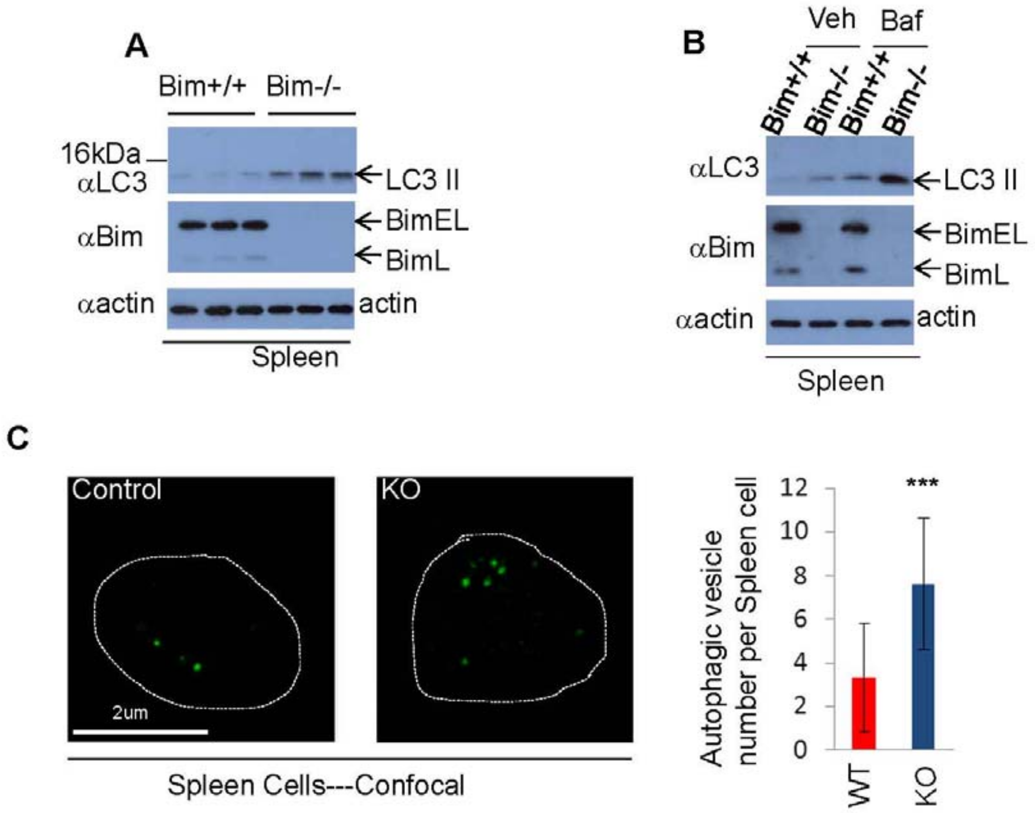

Figure S3. Autophagy Is Increased in *Bim* Knockout Mice, Related to Figure 3

(A) Spleen cells from wild-type (*Bim*$^{+/+}$) mice and *Bim* knockout (*Bim*$^{-/-}$) mice were cultured in RPMI 1640 with 10% FBS for 24 hours. Cell lysates were subjected to SDS-PAGE and immunoblotting with anti-LC3 antibody. Samples were from three knockout and control mice.

(B) Spleen cells from *Bim*$^{+/+}$ and *Bim*$^{-/-}$ mice were cultured in RPMI 1640 with 10% FBS for 24 hours, and treated with DMSO (vehicle) or Baf for 4 hours. Cell lysates were subjected to SDS-PAGE and immunoblotting with anti-LC3 antibody.

(C) Spleen cells from *Bim*$^{+/+}$ and *Bim*$^{-/-}$ mice were cultured in RPMI 1640 for 24 hours. Cells were then fixed with 4% PFA and stained with rabbit anti-LC3 antibody. Images showing examples of vesicles in spleen cells, were acquired under a confocal microscope. Autophagic vesicles were scored under a fluorescent microscope. Approximately 100 cells were counted in multiple random visual fields per sample. The figures show representative data from experiments in triplicate (n=90-100). Data are shown as mean±sd. ***: P<0.0001.

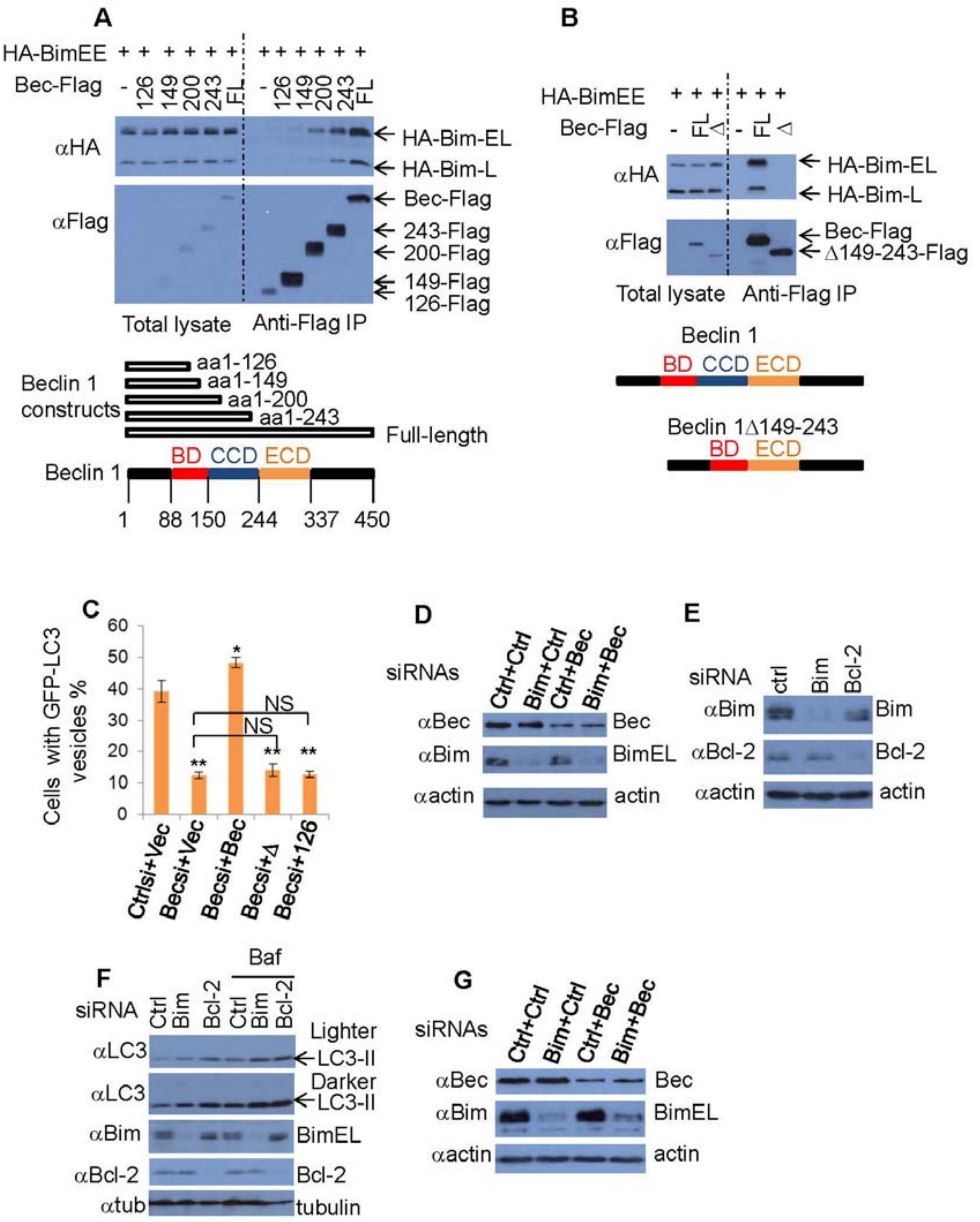

Figure S4. The Effect of Bim on Autophagy Is Beclin 1-Dependent, Related to Figure 4
(A) HA-BimEE/empty vector (IP negative control), HA-BimEE/Beclin 1-1-126aa-Flag (126), HA-BimEE/Beclin 1 1-149aa-Flag (149), HA-BimEE/Beclin 1 1-200aa-Flag (200), HA-BimEE/Beclin 1 1-243aa-Flag (243), or HA-BimEE/full-length Beclin 1-Flag (FL) were transfected into HeLa cells. After 20 hours, cells were then lysed and anti-Flag antibody was used for immunoprecipitation. Immunoprecipitates and total lysates were probed with anti-HA, anti-Flag, antibodies. BD: Bcl-2 binding domain; CCD: coiled-coil domain; ECD: evolutionary conserved domain (Furuya et al., 2005).

(B) HA-BimEE/empty vector (IP negative control), HA-BimEE/Beclin 1-Flag (FL) or HA-BimEE/Beclin 1-Δ149-243aa-Flag (Δ) were transfected into HeLa cells. After 20 hours, cells were then lysed and anti-Flag antibody was used for immunoprecipitation. Immunoprecipitates and total lysates were probed with anti-HA, anti-Flag antibodies.

(C) HeLa cells were transfected with control siRNA or Beclin 1 siRNA. After 24 hours, cells were split. After further 16 hours, the cells were transfected with vector GFP-LC3/vector (1:3), or GFP-LC3/Beclin 1, GFP-LC3/Beclin 1 Δaa149-243 (Δ), GFP-LC3/Beclin 1 aa1-126 (126), as indicated. After 20 hours, cells were fixed. The percentages of cells with GFP-LC3 vesicles were assessed. Data are shown as mean±sd. **: $P<0.01$; *: $P<0.05$; NS: not significant.

(D) HeLa cells were treated with control siRNA, Bim siRNA+control siRNA, control siRNA+Beclin 1 siRNA, or Bim siRNA+Beclin 1 siRNA, in parallel to Fig 4B. After 48 hours, cells were then harvested and were subjected to SDS-PAGE and blots were probed with Beclin 1, Bim, and actin antibodies.

(E) GFP-mRFP-LC3 stably expressing HeLa cells were treated with control siRNA, Bim siRNA or Bcl-2 siRNA, in parallel to Fig 4C. After 48 hours, cells were then harvested and were subjected to SDS-PAGE and blots were probed with Bim, Bcl-2 and actin antibodies.

(F) HeLa cells were treated with control siRNA, Bim siRNA or Bcl-2 siRNA. After 48 hours, one set of transfections were treated with Baf for 4 hours. Cells were then harvested and were subjected to SDS-PAGE and blots were probed with anti-LC3, Bim, Bcl-2 and tubulin antibodies.

(G) GFP-mRFP-LC3 cells were treated with control siRNA, Bim siRNA+control siRNA, control siRNA+Beclin 1 siRNA, or Bim siRNA+Beclin 1 siRNA, in parallel to Fig 4D. After 48 hours, cells were then harvested and were subjected to SDS-PAGE and blots were probed with Beclin 1, Bim and actin antibodies.

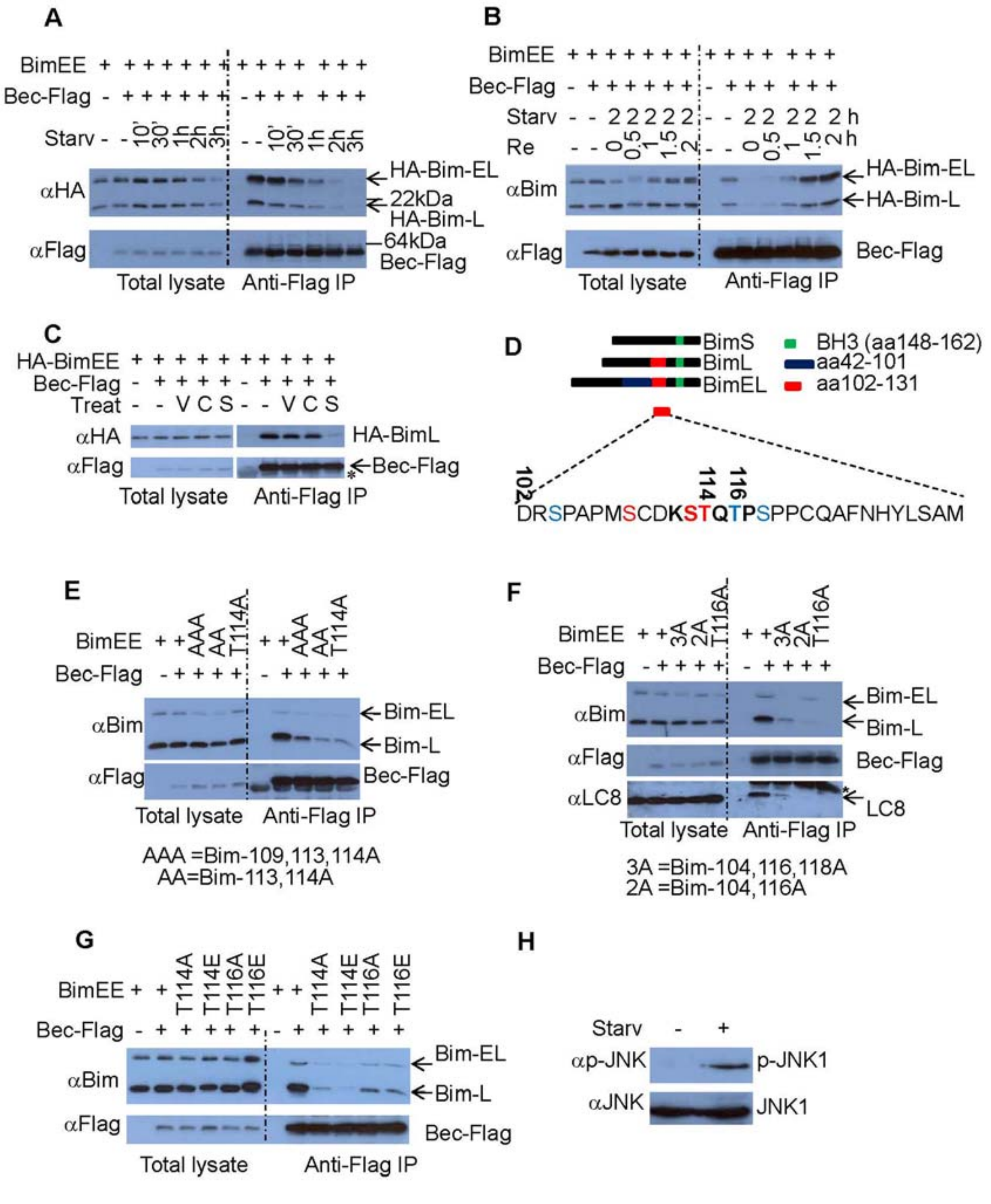

Figure S5. Starvation-Induced BimT116 Phosphorylation Disrupts the Bim-Beclin 1 Interaction, Related to Figure 5

(A) Time course of the Bim-Beclin 1 interaction after starvation. HA-Bim(EL)EE/vector (IP negative control), or HA-Bim(EL)EE/Beclin 1-Flag (6 replicates) were transfected into HeLa cells. After 24 hours, HA-Bim(EL)EE/Beclin 1-transfected cells underwent HBSS (Hank's-Balanced Salt Solution) starvation (Starv) for 0 (no starvation), 10min, 30min, 1h, 2h, 3h, respectively. The cells were lysed and anti-Flag (M2) was used for immunoprecipitation. Immunoprecipitates and total lysates were detected with anti-Flag (Rabbit) and anti-HA.

(B) Serum restimulation restores starvation-reduced Beclin 1-Bim interaction. HA-Bim(EL)EE/vector (IP negative control) or HA-Bim(EL)EE/Beclin 1-Flag (6 replicates) were transfected into HeLa cells. After 24 hours, HA-Bim(EL)EE/Beclin 1-transfected cells underwent HBSS starvation for 0 (no starvation, 1 of the replicates), 2h (5 of the replicates), respectively. The starved cells were restimulated (Re) with serum-containing media for 0, 0.5h, 1h, 1.5h, 2h, respectively. The cells were lysed and anti-Flag (M2) was used for immunoprecipitation. Immunoprecipitates and total lysates were detected with anti-Flag (Rabbit) and anti-HA.

(C) HA-Bim(L)EE/empty vector (IP negative control), HA-Bim(L)EE/Beclin 1-Flag were transfected into HeLa cells. After 20 hours, one set of HA-Bim(L)EE transfection was treated with 1uM verapamil (V) (Sigma) for 8 hours, one set of HA-Bim(L)EE transfection was treated with 20 uM calpastatin (C) peptide (Calbiochem) for 8 hours, and one set of HA-Bim(L)EE transfection was starved (S) in HBSS for 2 hours. Cells were then lysed and anti-Flag antibody was used for immunoprecipitation. Immunoprecipitates and total lysates were probed with anti-HA, anti-Flag (M2) antibodies.

(D) The potential phosphorylation sites in Bim aa102-131 (the region that differs between BimL and BimS). The letters in blue and red represent 6 potential phosphorylation sites. Note that the LC8 binding motif KSTQTP in Bim is located in the region that differs between Bim-L and BimS (in red).

(E) Bim T114 is important for the Bim-Beclin 1 interaction. Bim(EL)EE/vector (IP negative control), Bim(EL)EE/Beclin 1-Flag, Bim(EL)EE-AAA (S109A, S113A, T114A, letters in red in (D))/Beclin 1-Flag, Bim(EL)EE-AA (S113A, T114A)/Beclin 1-Flag or Bim(EL)EE-T114A/Beclin 1-Flag were transfected into HeLa cells. After 20 hours, the cells were lysed and anti-Flag (M2) was used for immunoprecipitation. Immunoprecipitates and total lysates were detected with anti-Flag (M2) and anti-Bim.

(F) Bim T116 is important for the Bim-Beclin 1 interaction. Bim(EL)EE/vector (IP negative control), Bim(EL)EE/Beclin 1-Flag, Bim(EL)EE-3A (S104A, T116A, S118A, letters in blue in (D))/Beclin 1-Flag, Bim(EL)EE-2A (S104A, T116A)/Beclin 1-Flag or Bim(EL)EE-T116A/Beclin 1-Flag were transfected into HeLa cells. After 20 hours, the cells were lysed and anti-Flag (M2) was used for immunoprecipitation. Immunoprecipitates and total lysates were detected with anti-Flag (Rabbit), anti-Bim and anti-LC8. Note that the levels of endogenous LC8 pulled down by Beclin 1 correlated with the strength of Bim-Beclin 1 association. *: truncated Beclin 1-Flag (enriched by IP) band remaining after stripping.

(G) Mutations in T114 or T116 of Bim weaken the Bim-Beclin 1 interaction. Bim(EL)EE/vector (IP negative control), Bim(EL)EE/Beclin 1-Flag, Bim(EL)EE-T114A/Beclin 1-Flag, Bim(EL)EE-T114E (Bim T114 phospho-mimic)/Beclin 1-Flag, Bim(EL)EE-T116A/Beclin 1-Flag or Bim(EL)EE-T116E (Bim T116 phospho-mimic)/Beclin 1-Flag were transfected into HeLa cells. After 20 hours, the cells were lysed and anti-Flag (M2) was used for immunoprecipitation. Immunoprecipitates and total lysates were detected with anti-Flag (Rabbit), anti-Bim.

(H) HeLa cells were cultured with no starvation (in full medium) or 2-hour nutrient starvation (HBSS). The cell lysates were resolved by SDS-PAGE and probed with phospho-SAPK/JNK (Thr183/Tyr185) and anti-total JNK antibodies.

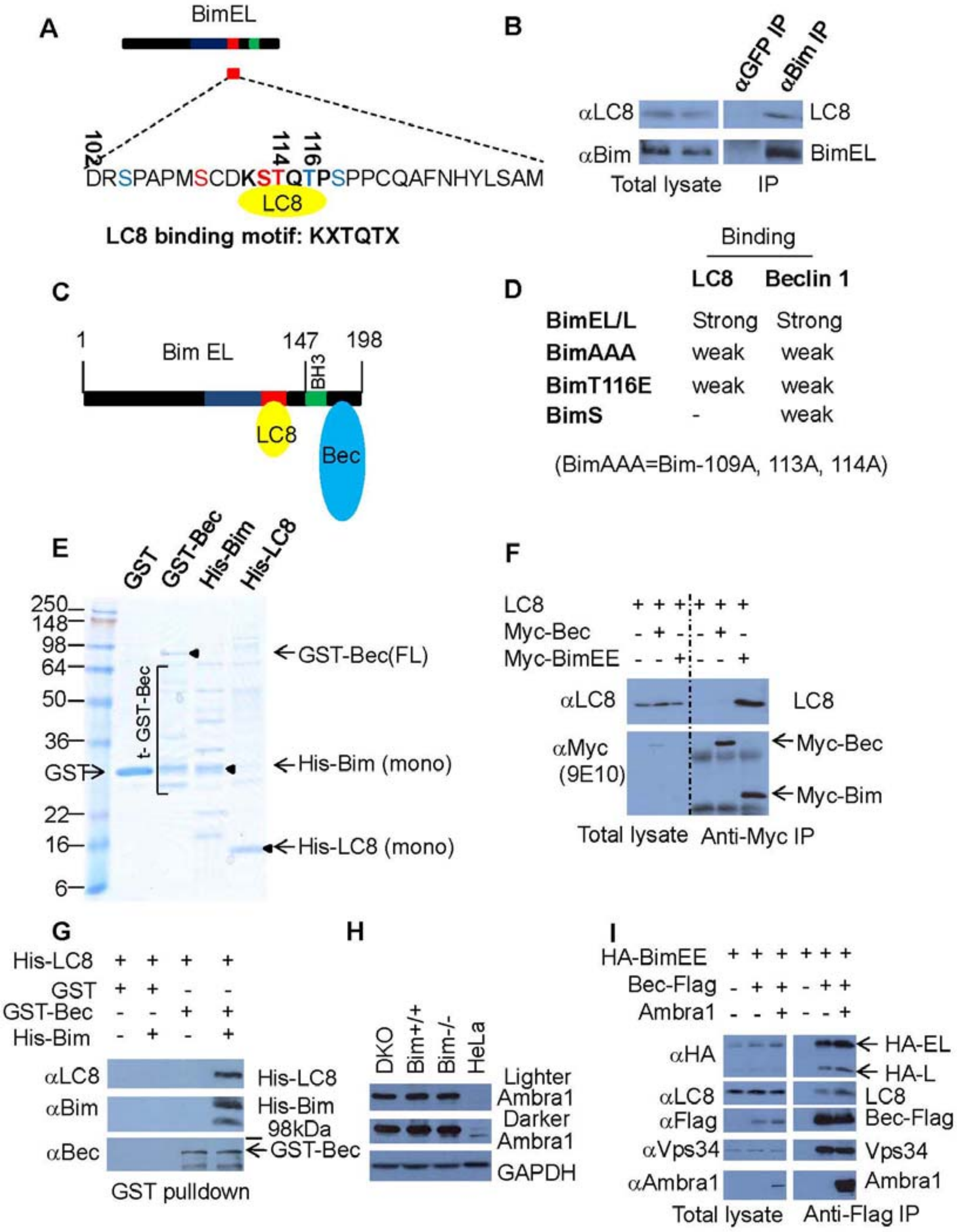

Figure S6. LC8 Promotes the Bim-Beclin 1 Interaction, Related to Figure 6

(A) The consensus LC8 binding motif KXTQTX in Bim. The LC8 binding motif KSTQTP in Bim is located in the region that differs between Bim-L and Bim-S (in red). The letters in blue and red represent 6 potential phosphorylation sites. Note that T114 and T116 are essential in the consensus motif for LC8 binding.

(B) HeLa cells were lysed and subjected to anti-GFP antibody (Rabbit polyclonal) (negative control) and anti-Bim antibody (Rabbit polyconal) immunoprecipitation. Immunoprecipitates and total lysates were resolved by SDS-PAGE and probed with anti-LC8 and anti-Bim.

(C) Diagram of Bim and its binding domains for LC8 and Beclin 1. It is known that AA112-131 within Bim (in red) binds to LC8. Based on data from Figure 6A-B, we propose that the C-terminal (beyond the BH3 domain) of Bim binds to Beclin 1.

(D) The binding "affinities" of Bim variants to LC8 and Beclin 1 (based on Figure 6C-E and Figure 1J).

(E) His-Bim, His-LC8 and GST-Beclin 1 and GST were expressed in BL21(DE3) E.coli. GST and GST-Beclin 1 were purified with glutathione sepharose. Full-length (FL) GST-Beclin 1 (GST-Bec) is marked with an arrow head (truncated GST-Beclin 1 is labelled as t-GST-Bec). His-LC8 and His-Bim were purified with nickel-charged His-bind resin. Full-length monomeric (mono) His-Bim and His-LC8 are marked with arrow heads.

(F) LC8 does not interact with Beclin 1, while LC8 interacts with Bim. LC8/vector (IP negative control), LC8/Myc-Bim(EL)EE, or LC8/Myc-Bec were transfected into HeLa cells. After 20 hours, the cells were lysed and anti-Myc (Rabbit) was used for immunoprecipitation. Immunoprecipitates and total lysates were detected with anti-LC8 (Rabbit) and anti-Myc (9E10).

(G) His-Bim, His-LC8 or GST-Beclin 1 were expressed in BL21(DE3) E.coli and purified. 2ug His-LC8 was combined with 2ug GST without or with 1ug His-Bim (as controls); 2ug His-LC8 was combined with 2ug GST-Beclin 1 without or with 1ug His-Bim. The mixtures were incubated in Buffer A for 3 hours. Glutathione beads were then used to pull down GST or GST-Beclin 1. The pull-down products were detected with anti-LC8, anti-Bim and anti-Beclin 1.

(H) The lysates of *Bax/Bak* DKO, *Bim*$^{+/+}$, *Bim*$^{-/-}$ MEFs, or HeLa cells were subjected to SDS-PAGE and subjected to immunoblotting with anti-Ambra1 and GAPDH antibodies.

(I) HA-BimEE/empty vectors (IP negative control), HA-BimEE/Beclin 1-Flag/empty vector, or HA-BimEE/Beclin 1-Flag/Ambra1 were transfected into HeLa cells. After 20 hours, cells were then lysed and anti-Flag antibody was used for immunoprecipitation. Immunoprecipitates and total lysates were probed with anti-HA, anti-LC8, anti-Flag, Vps34, and anti-Ambra1antibodies.

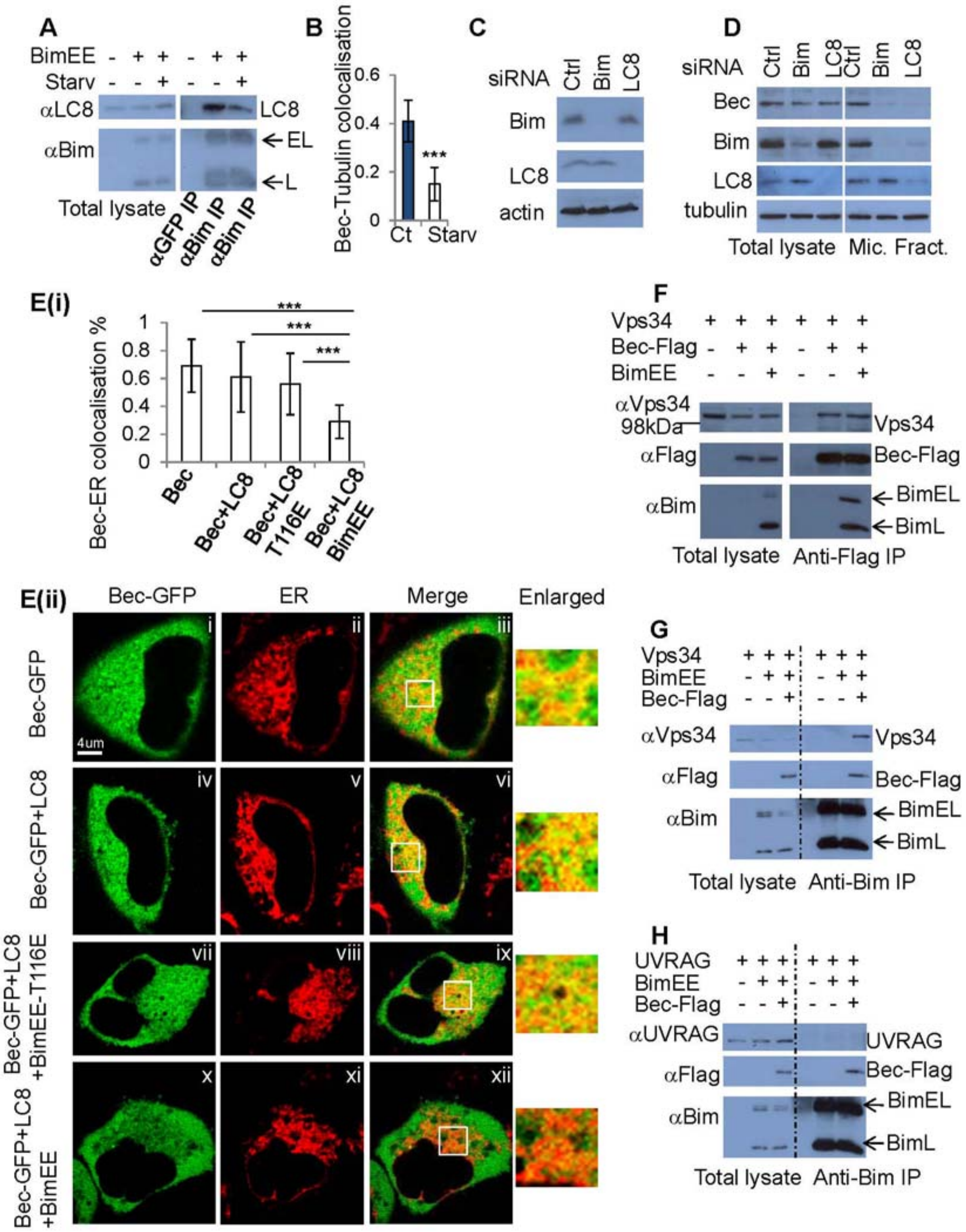

Figure S7. Bim Inactivates Beclin 1 by Confining It to Microtubules, Related to Figure 7
(A) HeLa cells were transfected with vector or Bim(EL)-EE (2 replicates). After 20 hours, one set of cells with Bim(EL)-EE were starved in HBSS for 2 hours. The cell lysates were subjected to anti-GFP antibody (Rabbit polyclonal) (negative control) and anti-Bim antibody (Rabbit polyconal) immunoprecipitation. Immunoprecipitates and total lysates were resolved by SDS-PAGE and probed with anti-LC8 and anti-Bim.

(B) Colocalisations (for Fig 7D) were quantified from images in 15 cells with Volocity program (Colocalization coefficient Mx). Ct: control, Starv: Starve. Data are shown as mean±sd. ***: $P<0.001$.

(C) Control siRNA, Bim siRNA and LC8 siRNA were transfected to cells in parallel to Fig 7F. After 48 hours, cells were harvested and subjected to immunoblotting with anti-Bim, anti-LC8 and anti-actin antibodies.

(D) HeLa cells were treated with control siRNA, Bim siRNA, or LC8 siRNA for 48 hours. Cells were then harvested and microtubules were fractionated. Microtubule fractions and total lysates were subjected to SDS-PAGE and detected with anti-Beclin 1, anti-Bim, anti-LC8 and anti-tubulin antibodies.

(E) Beclin 1-GFP/empty vectors, Beclin 1-GFP-Myc-LC8/empty vector, Beclin 1-GFP-Myc-LC8-Bim(EL)EE T116E, or Beclin 1-GFP-Myc-LC8-Bim(EL)EE were transfected into HeLa cells. After 20 hours, cells were stained with ER tracker. Live images were taken under confocal microscope. Colocalisations were quantified from images from 10-15 cells with Volocity program (Colocalization coefficient Mx). Data are shown as mean±sd. ***: $P<0.001$.

(F) Vps34/empty vectors (IP negative control), Vps34/Beclin 1-Flag/vector or Vps34/Beclin 1-Flag/Bim(EL)EE were transfected into HeLa cells. After 20 hours, the cells were lysed and anti-Flag (M2) was used for immunoprecipitation. Immunoprecipitates and total lysates were detected with anti-Vps34, anti-Flag (Rabbit) and anti-Bim.

(G) Vps34/empty vectors (IP negative control), Vps34/Beclin 1-Flag/empty vector, or Vps34/Beclin 1-Flag/Bim-EE were transfected into HeLa cells. After 20 hours, cells were then lysed and anti-Bim antibody was used for immunoprecipitation. Immunoprecipitates and total lysates were probed with anti-Vps34, anti-Flag (Beclin 1), and anti-Bim antibodies.

(H) UVRAG/empty vectors (IP negative control), UVRAG/Beclin 1-Flag/empty vector, or UVRAG/Beclin 1-Flag/Bim-EE were transfected into HeLa cells. After 20 hours, cells were then lysed and anti-Bim antibody was used for immunoprecipitation. Immunoprecipitates and total lysates were probed with anti-UVRAG, anti-Flag (Beclin 1), and anti-Bim antibodies.

SUPPLEMENTAL REFERENCES

Bouillet, P., Metcalf, D., Huang, D.C., Tarlinton, D.M., Kay, T.W., Kontgen, F., Adams, J.M., and Strasser, A. (1999). Proapoptotic Bcl-2 relative Bim required for certain apoptotic responses, leukocyte homeostasis, and to preclude autoimmunity. Science *286*, 1735-1738.

Chen, L., Willis, S.N., Wei, A., Smith, B.J., Fletcher, J.I., Hinds, M.G., Colman, P.M., Day, C.L., Adams, J.M., and Huang, D.C. (2005). Differential targeting of prosurvival Bcl-2 proteins by their BH3-only ligands allows complementary apoptotic function. Mol Cell *17*, 393-403.

Ewings, K.E., Hadfield-Moorhouse, K., Wiggins, C.M., Wickenden, J.A., Balmanno, K., Gilley, R., Degenhardt, K., White, E., and Cook, S.J. (2007). ERK1/2-dependent phosphorylation of BimEL promotes its rapid dissociation from Mcl-1 and Bcl-xL. EMBO J *26*, 2856-2867.

Furuya, N., Yu, J., Byfield, M., Pattingre, S., and Levine, B. (2005). The evolutionarily conserved domain of Beclin 1 is required for Vps34 binding, autophagy and tumor suppressor function. Autophagy *1*, 46-52.

Hubner, A., Barrett, T., Flavell, R.A., and Davis, R.J. (2008). Multisite phosphorylation regulates Bim stability and apoptotic activity. Mol Cell *30*, 415-425.

Luo, S., and Rubinsztein, D.C. (2010). Apoptosis blocks Beclin 1-dependent autophagosome synthesis: an effect rescued by Bcl-xL. Cell Death Differ *17*, 268-277.

Luo, S., Vacher, C., Davies, J.E., and Rubinsztein, D.C. (2005). Cdk5 phosphorylation of huntingtin reduces its cleavage by caspases: implications for mutant huntingtin toxicity. J Cell Biol *169*, 647-656.

Pattingre, S., Tassa, A., Qu, X., Garuti, R., Liang, X.H., Mizushima, N., Packer, M., Schneider, M.D., and Levine, B. (2005). Bcl-2 antiapoptotic proteins inhibit Beclin 1-dependent autophagy. Cell *122*, 927-939.

Schinzel, A., Kaufmann, T., Schuler, M., Martinalbo, J., Grubb, D., and Borner, C. (2004). Conformational control of Bax localization and apoptotic activity by Pro168. J Cell Biol *164*, 1021-1032.

Schmelzle, T., Mailleux, A.A., Overholtzer, M., Carroll, J.S., Solimini, N.L., Lightcap, E.S., Veiby, O.P., and Brugge, J.S. (2007). Functional role and oncogene-regulated expression of the BH3-only factor Bmf in mammary epithelial anoikis and morphogenesis. Proc Natl Acad Sci U S A *104*, 3787-3792.

Stewart, S.A., Dykxhoorn, D.M., Palliser, D., Mizuno, H., Yu, E.Y., An, D.S., Sabatini, D.M., Chen, I.S., Hahn, W.C., Sharp, P.A., *et al.* (2003). Lentivirus-delivered stable gene silencing by RNAi in primary cells. RNA *9*, 493-501.

Tanida, I., Yamaji, T., Ueno, T., Ishiura, S., Kominami, E., and Hanada, K. (2008). Consideration about negative controls for LC3 and expression vectors for four colored fluorescent protein-LC3 negative controls. Autophagy *4*, 131-134.

Winslow, A.R., Chen, C.W., Corrochano, S., Acevedo-Arozena, A., Gordon, D.E., Peden, A.A., Lichtenberg, M., Menzies, F.M., Ravikumar, B., Imarisio, S., *et al.* (2010). {alpha}-Synuclein impairs macroautophagy: implications for Parkinson's disease. J Cell Biol *190*, 1023-1037.
